# Effects of Social Housing Changes on Immunity and Vaccine-Specific Immune Responses in Adolescent Male Rhesus Macaques

**DOI:** 10.3389/fimmu.2020.565746

**Published:** 2020-10-15

**Authors:** Bapi Pahar, Kate C. Baker, Alexandra N. Jay, Kasi E. Russell-Lodrigue, Sudesh K. Srivastav, Pyone Pyone Aye, James L. Blanchard, Rudolf P. Bohm

**Affiliations:** ^1^Division of Comparative Pathology, Tulane National Primate Research Center, Covington, LA, United States; ^2^Division of Veterinary Medicine, Tulane National Primate Research Center, Covington, LA, United States; ^3^Department of Biostatistics, Tulane University, New Orleans, LA, United States

**Keywords:** housing, immunity, measles, neutralizing antibodies, rhesus macaque, T cells, B cells

## Abstract

Nonhuman primates (NHPs) in research institutions may be housed in a variety of social settings, such as group housing, pair housing or single housing based on the needs of studies. Furthermore, housing may change over the course of studies. The effects of housing and changes in housing on cell activation and vaccine mediated immune responses are not well documented. We hypothesized that animals moved indoors from group to single housing (GH-SH) would experience more stress than those separated from groups into pair housing (GH-PH), or those placed briefly into pair housing and separated 5 weeks later into single housing (GH-PH-SH). We also compared the effects of separation from group to pair housing with the separation from pair to single housing. Eighteen male rhesus macaques were followed over the course of changes in housing condition over 10–14 weeks, as well as prior to and after primary vaccination with a commercially available measles vaccine. We identified two phenotypic biomarkers, namely total CD8 population and proliferating B cells, that differed significantly across treatment groups over time. At 10 weeks post-separation, levels of proliferating B cells were higher in GH-SH subjects compared to GH-PH subjects, and in the latter, levels were lower at 10 weeks than prior to removal from group housing. At 2 weeks post-separation from group to single housing, the frequency of CD8+ T cells was higher in GH-SH subjects compared to one week post separation from pair into single housing in the GH-PH-SH subjects. Comparing the same elapsed time since the most recent separation activated CD20 populations were persistently higher in the GH-SH animals than the GH-PH-SH animals. Housing configuration did not influence vaccine-mediated responses. Overall, our study found benefits of pair housing over single housing, suggesting that perturbations in immune function will be more severe following separation from group to single housing than from pair to single housing, and supporting the use of short-duration pair housing even when animals must subsequently be separated. These findings are useful for planning the housing configurations of research NHPs used for vaccine studies and other studies where immune response is being assessed.

## Introduction

It has become increasingly apparent that stress significantly affects immune function in both humans and animals (1). The effect of a stressor depends on several factors, including but not limited to sex, age, duration, and the type of stressor experienced (2). In research animals, the interaction of stress and the immune system is an important physiologic mechanism to safeguard from spontaneous disease and is also a consideration in research design to assure the preservation of normal immune function, on which the validity of research results may depend. In nonhuman primates (NHPs), stress may occur due to routine husbandry practices or facility maintenance as well as any number of unavoidable consequences of animal housing (3).

Prior research has shown that common experimental conditions such as housing changes have a profound effect on immune function, disease progression, and survival times of rhesus macaques assigned to infectious disease studies (4, 5). It has long been recognized that removal from outdoor group housing to indoor cage housing is a significant source of stress to NHPs, one that results in immune perturbations (6–11). When these removals are implemented for the purpose of enrolling NHPs in research, the implications are significant and broadly apply to NHP biomedical research, potentially posing confounds to studies involving topics such as infectious disease pathogenesis, drug discovery and vaccine development. It is therefore important to remove NHPs from groups in a manner that minimizes the stress response and maximizes the stability of immune function.

Animals may be removed from large social groups into indoor caging for assignment to studies in a number of ways, including placement into familiar pairs or into single housing. For research projects requiring single housing, an initial period of pair housing may be feasible, with the aim of buffering the stress associated with group removal and accelerating the adjustment to indoor cage housing. The following study was designed to evaluate the effects of changes in these macroenvironmental and social housing conditions necessary for transition from outdoor social housing in a breeding colony to indoor housing for research and the effect on activated and proliferative T and B cell population on vaccine mediated immune responses. We hypothesize that:

1. The separation from group to single housing will result in greater stress than the separation from group housing to pair housing.
2. Ten weeks after removal from group housing, animals moved directly into single housing will display signs of greater stress than those moved either into long-term pairing or those that were placed into pair housing upon removal but subsequently separated into single housing.
3. The separation from group to single housing will result in greater levels of stress than the separation from pair to single housing.
4. Animals moved from group housing to pair housing will show stronger and more persistent vaccine-specific immune responses than animals moving to single housing, and animals moved from group housing to single housing with an initial 2-week post-separation period of pair housing will show stronger vaccine-specific immune responses than animals moved directly to single housing.

Changes in housing condition, which have the potential to induce stress, were monitored by measuring changes in immune system parameters in a longitudinal study of adolescent male rhesus macaques. We evaluated the effects of these changes on the frequency of different cellular activation, proliferation and other phenotypic markers, and immune responses to measles vaccination to determine the social management that best supports increased vaccine specific immune responses and regulation of B and T cell activation and proliferation.

## Materials and Methods

### Animals and Housing

Eighteen healthy Indian ancestry male rhesus macaques (*Macaca mulatta*), aged 3.8 to 4.3 years of age, were assigned to this study. Subjects were born in the specific pathogen free breeding colony at the Tulane National Primate Research Center (TNPRC). All subjects were antibody and virus-negative for simian retrovirus Type D and seronegative for simian T-lymphotropic virus I, simian immunodeficiency virus, *Macacine herpesvirus* I, simian varicella virus, and measles virus. In addition, animals were negative for *Mycobacterium tuberculosis*. The study was approved by the TNPRC Institutional Animal Care and Use Committee (IACUC) and was conducted within the guidelines of the United States Public Health Service Policy and the *Guide for the Care and Use of Laboratory Animals* (12). TNPRC has full accreditation by AAALAC.

**Figure 1:**
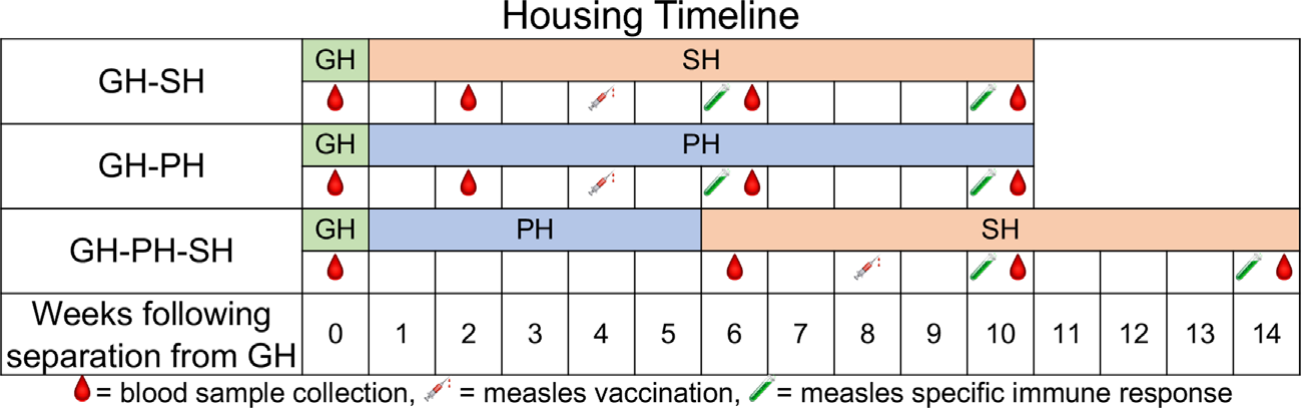
Housing conditions and measles vaccination schedule for treatment groups over the duration of the study are shown. The housing conditions include GH-SH (group housed to single housed), GH-PH (group housed to paired housed), and GH-PH-SH (paired housed to single housed) along-with the sample collection timepoints for each group. All animals were group-housed prior to initiation of the study. Two and six weeks post-vaccination time points were used for measuring antigen specific T cell responses and measles specific neutralizing antibody titers in macaques.

All animals were group housed in outdoor field cages and subsequently moved to indoor caging, either in familiar pairs, singly, or initially in familiar pairs and later separated into single housing (**Figure 1**). Outdoor housing enclosures were field cages constructed of wire mesh walls and roof, with natural/earthen ground cover flooring and a variety of furnishings including perches, shelters, and swings. Field cages were populated with 11–29 rhesus macaques of mixed sex and ages. Water was available ad libitum and a standard, commercially formulated NHP diet (Purina Diet 5037, PMI Feeds, St. Louis, MO) was provided daily and supplemented with fresh fruit and/or forage material no fewer than three times per week as part of the TNPRC behavioral management program. All macaques were examined approximately twice a year during semiannual health assessments (SAHAs). Routine examination and preventative medicine treatments (vaccines, anthelmintics) are administered during SAHA and blood is collected for diagnostic purposes or per an IACUC approved protocol for research. One such preventative measure is vaccination for measles, a highly contagious and potentially fatal viral disease in rhesus macaques characterized by pyrexia, pneumonia, maculopapular rash, and death (13, 14). All animals assigned to this study had not previously been vaccinated for measles.

Indoors, subjects were maintained in Animal Biosafety Level 2 housing with a 12:12-h light/dark cycle, relative humidity of 30%–70%, and a temperature of 64°F–72°F (17.8°C to 22.2°C). Water and food were provided to indoor housed animals similarly to the animals in outdoor housing except that feeding enrichment was distributed five times per week. Animals were housed in stainless steel cages (Allentown, Inc., Allentown, NJ) sized in excess of the U.S. Department of Agriculture (USDA) regulations; each cage contained a perch, portable enrichment toy, and a forage board for feeding enrichment. Once moved inside, all subjects were housed in the same room and had visual and auditory access to conspecifics for the duration of the study. The arrangement of caging in the indoor animal housing room remained unchanged throughout the study.

### Study Design and Procedures

Subjects were enrolled and assigned to three treatment groups, each experiencing different housing conditions over a period of 14 weeks (**Figure 1**). The housing conditions examined were as follows: animals removed from group housing and placed into single housing (GH-SH), animals removed from group housing and placed into familiar pairs which remained together for the course of the study (GH-PH), and animals removed from group housing, placed into familiar pairs for 5 weeks, and subsequently separated into single housing (GH-PH-SH). These groups were chosen because they represent the various housing changes associated with the removal of animals from the breeding colony for assignment to research projects at the TNPRC.

All animals were vaccinated for measles using a commercially available canine distemper/measles vaccine (CDMV, Vanguard @DM, Zoetis) which has previously been shown to be protective in rhesus macaques (15). One ml of reconstituted vaccine was administered by the intramuscular route 4 weeks after removal from GH for the GH-PH and GH-SH groups. In the GH-PH-SH group, it was administered 3 weeks following pair separation (8 weeks after removal from group housing). (**Figure 1**). Samples for animals in all groups were collected as depicted in **Figure 1**.

Animals were accessed for venipuncture under ketamine anesthesia (10 mg/kg) administered by IM injection. Peripheral blood (PB) was used for T and B cell immuno-phenotyping, antigen-specific intracellular cytokine flow cytometry (CFC) assay in T cells, and complete blood counts (CBC). Serum/plasma samples were used for measles antibody titer analyses.

### Preparation of Canine Distemper and Measles Vaccine (CDMV) Antigen

Vero cells were grown in minimal essential medium (BioWhittaker, MD) with 10% fetal calf serum and 1% L-glutamine and infected with modified live CDMV at a low multiplicity of infection. Viruses were harvested at 6–7 days (80%–90% cytopathic effect), sonicated, freeze/thawed three times, centrifuged, and supernatant was collected and used as an antigen for intracellular CFC assay as reported earlier (16, 17).

### T and B Cell Phenotyping by Flow Cytometry Staining

Lymphocytes from PB were isolated by density gradient centrifugation (Lymphocyte Separation Medium, Cellgro, MA) (18, 19). T and B-lymphocytes were quantified for their regulatory function (CD25), activation (CD38+, CD69+, HLA-DR+), proliferation (Ki67+), trafficking markers (CD62L+), and naive/memory distribution (CD28+ and CD95+). For cell surface staining, 100 μl of EDTA blood was stained with directly conjugated monoclonal antibodies (MAbs) using a whole blood lysis staining protocol (19–21). Cells were first stained with live/dead stain (Invitrogen). MAbs used for this study were anti-CD3, anti-CD4, anti-CD8, anti-CD62L, anti-CD95, anti-HLA-DR, anti-CD25 and anti-CD69, anti-CD38, anti-CD20, and anti-Ki67 antibodies (**Supplemental Table S1**). For detecting Ki67+ proliferating cells, the intracellular staining protocol was performed as reported earlier (22, 23). After staining cells were fixed in BD Stabilizing and fixative buffer and at least 30,000 events were collected from each sample by lymphocyte gating and the data were analyzed with FlowJo software (TreeStar, Ashland, OR).

### Intracellular CFC Assay

To quantify cytokine production in CD4+ and CD8+ T cells in response to CDMV antigen specific stimulation, a CFC assay was used on freshly isolated Na heparinized PB mononuclear cells according to methods previously described (16). Briefly, PB mononuclear cells were resuspended at 1 × 10^7^ cells/ml in RPMI 1640 medium supplemented with 10% fetal calf serum and penicillin/streptomycin. In addition, 1 × 10^6^ PBMCs/400 μl was placed in a 48-well flat-bottomed plate (BD Biosciences) and were stimulated with antigen. Control cells were stimulated with either media (negative control) or staphylococcal enterotoxin B (positive control), 200 ng/ml (SEB, Toxin Technology, Sarasota, FL), respectively.

For CDMV antigen stimulation, 40 μl of lysed CDMV virions was used. Co-stimulatory antibodies, anti-CD28 and anti-CD49d, were added at 1 μg/ml (**Supplemental Table S1**). The cultures were incubated at 37°C in a humidified 5% CO_2_ atmosphere for 6 h. Brefeldin A (10 μg/ml, Sigma) was added after 1 h. After incubation, the 48-well plate was transferred to a refrigerator overnight. All cells were transferred to a polypropylene tube and washed once with 3 ml of cold PBS with 0.1% BSA (Sigma) and 7 mM sodium azide (Sigma) (PBS/BSA) as reported earlier (21). The cells were stained first for live/dead stain followed by anti-CD3, CD4, and CD8 MAbs. Following washing and fixation/permeabilization, the cells were stained for intracellular markers using anti-IFNγ, TNFα, and IL-10 MAbs (**Table S1**). Isotype-matched control antibodies were used to confirm the staining specificity. After washing, the cells were resuspended in 1× BD stabilizing and fixative buffer. Data were acquired within 24 h of staining using a BD Fortessa instrument. Cells were gated on singlets, lymphocytes, followed by live cells and then on CD3+ T cells and subsequently on CD3+CD4+ and CD3+CD8+ T cell subsets. CD3+CD4+ or CD3+CD8+ T cells were further analyzed for the presence of IFNγ, TNFα, and IL-10 positive cells by using Flowjo software (**Supplemental Figure S1**). The criterion for a positive cytokine response was greater than 0.05% and a two-fold increase in frequency for that specific antigen and cytokine above the medium control culture. All positive values were subtracted from the values of medium control.

### Serum Neutralization Measles Antibody Titer

Plaque reduction microneutralization (PRMN) assay was performed to detect functional neutralizing antibodies in serum samples collected from all macaques at 2 and 6 weeks following vaccination as described previously (24, 25). In brief, 50 µl of serum (serially diluted from 1:20 to 1:640) was incubated with 50 µl of ~50 CCID_50_ measles virus MVvac2GFP, a molecular clone of the Moraten vaccine strain expressing green fluorescent protein at 37°C, with 95% humidity and 5% CO_2_ in a 96 well microtiter plate. After 1 h, 2 × 10^4^ low passage Vero cells were added to each well and incubated for 3 days. The wells were observed microscopically at 10X and scored for the presence (no neutralization) or absence (neutralization) of fluorescence. Negative cell controls to assess cell confluence and toxicity, as well as plaque positive (30%–75%) virus controls were also run. The data were analyzed using the Reed Muench equation to compute a reciprocal titer for each sample. To standardize and compare results, a neutralizing antibody score [(sample titer/rhesus measles immunoglobulin (MIG) control titer tested in parallel) × 100] was calculated for each sample.

### Statistical Analyses and Graphics

Analyses focus on comparing housing groups at two or more time points in order to determine the differences in immunological outcomes between different housing groups over time. Main effects of group and group-by-time effects were analyzed using Repeated Measures Analysis of Variance (rmANOVA) along with separate ANOVAs for each time point. Bonferroni’s multiple comparison test was used for comparing more than two groups in ANOVA tests for *post hoc* analysis. The significance of interaction between time and treatment group was assessed to discern the group differences at the linear or the quadratic terms over time. A two-sided 5% significance level was used throughout the analyses. All data analyses, summaries and listing were performed using SAS software (version 9.4 in a Windows environment). All graphics were plotted using Prism software (GraphPad software, San Diego, CA). Due to issues experienced with sample processing, data from 2 of 6 GH-SH animals and 2 of 6 GH-PH animals are absent at pre-separation time points (Baseline).

To determine the effect of housing change on different T and B cell phenotypes and vaccine mediated immune responses, comparisons were performed on samples from the following treatment groups and time points.

For hypothesis 1 (The separation from group to single housing will result in greater stress than the separation from group housing to pair housing), data from the GH-SH and GH-PH groups were compared between baseline (group housing prior to removal), 2 weeks following removal from group housing (animals housed either in pair or single housing), 6 weeks following removal, and 10 weeks following removal (**Figure 1**).

For hypothesis 2 (Ten weeks after removal from group housing, animals moved directly into single housing will display signs of greater stress than those moved either into long-term pairing or those that were placed into pair housing upon removal but subsequently separated), data from the GH-SH, GH-PH, and GH-PH-SH subjects were compared between the pre-separation time point and 10 weeks following removal from group housing.

For hypothesis 3 (The separation from group to single housing will result in greater levels of stress than the separation from pair to single housing), data from the GH-PH and GH-PH-SH groups were compared at the following time points: 1) the time point prior to removal from group housing for both treatment groups, 2) 2 weeks following removal from group housing for the GH-PH animals and 1 week after separation from pair to single housing in the GH-PH-SH subjects, 3) 6 weeks following separation from group housing in the GH-PH group and 5 weeks following separation from pair housing to single housing in the GH-PH-SH subjects, and 4) 10 weeks following separation from group housing in the GH-PH group and 9 weeks following separation from pair to single housing in the GH-PH-SH group (**Figure 1**).

For hypothesis 4 (Animals moved from group housing to pair housing will show stronger and more persistent vaccine-specific immune responses compared to animals moving to single housing, and animals moved from group housing to single housing with an initial 2-week post-separation period of pair housing will show stronger vaccine-specific immune responses than animals moved directly to single housing): 2- and 6-weeks post-measles vaccination time points were assessed (**Figure 1**).

Levels of immune response expression are reported for each treatment group *via* means, standard errors of the mean, and confidence intervals at each separate time point (See **Supplemental Tables S2–S4**).

## Results

### Effects of Removal From Groups Into Pair Housing Versus Single Housing on T and B Cell Population in Peripheral Blood (Hypothesis 1)

**Figure 2:**
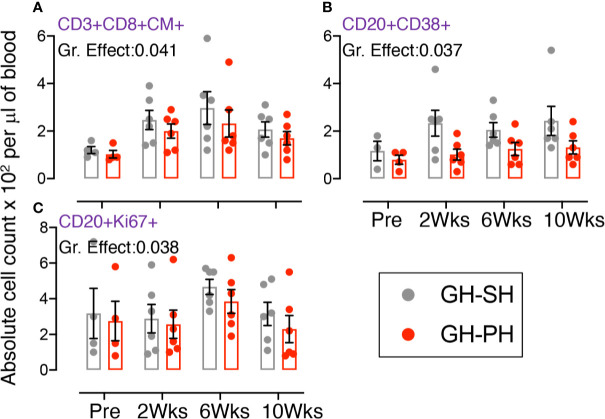
Absolute count of peripheral blood central memory (CM) CD3+CD8+CD28+CD95+, activating CD20+CD38+, and proliferating CD20+Ki67+ lymphocyte were shown for GH-SH and GH-PH groups. A significant difference in absolute CD8+CM+ **(A)** and CD20+CD38+ **(B)**, and CD20+Ki67+ **(C)** cells was detected between these groups across house treatment time points. The X axis denotes pre- and post-separation housing time points and the Y axis denotes absolute counts. Individual values from each animal are shown as colored circles with bars showing mean ± standard error for each group.

Twelve animals assigned to two treatment groups, GH-SH and GH-PH, were used for these analyses (**Table 1**, **Figure 2**, **Supplemental Tables S2–S4**, and **Supplemental Figures S2**-**S4**).

**Table 1:** Significant changes in the frequency of peripheral T and B cell subpopulations.

Groups#	Effect	Cell Phenotype		
		CD3+CD4+	CD3+CD8+	CD3-CD20+
Hypothesis 1: GH-SH vs. GH-PH	Group effect		CM+ (p = 0.041)	CD38+ (p = 0.037) Ki67+ (p = 0.038)
Hypothesis 2: GH-SH vs. GH-PH vs. GH-PH-SH	Group effect	CD4+ (p = 0.017) CD62L+ (p = 0.022) CM+ (p = 0.013) Naïve+ (p = 0.044) HLADR+ (p = 0.003) CD25+ (p = 0.005) EM+ (p = 0.023) CD38+ (p = 0.039)	CD8+ (p = 0.003) Naive+ (p = 0.021) CM+ (p = 0.002) Ki67+ (p = 0.009) CD69+ (p = 0.005) CD62L+ (p = 0.002) CD38+ (p = 0.002)	CD25+ (p = 0.016) CD38+ (p = 0.003) CD62L+ (p = 0.006)
	group x time effect			Ki67+ (p = 0.031)
Hypothesis 3: GH-SH vs. GH-PH-SH	Group effect	CD25+ (p = 0.003)	CD8+ (p = 0.018) CM+ (p = 0.003) Ki67+ (p = 0.043) CD62L+ (p = 0.003)	Ki67+ (p = 0.030)
	group x time effect		CD8+ (p = 0.026)	

^#^ For GH-SH to GH-PH groups, data were compared at pre (0), 2, 6, and 10 weeks post-separation from GH (see more detail in **Figure 1**).

^#^ For GH-SH, GH-PH and GH-PH-SH groups, data were compared at pre (0) and 10 weeks post-separation from GH (see more detail in **Figure 1**).

^#^ For GH-SH to GH-PH-SH groups, data were compared between GH-SH at pre (0), 2, 6, and 10 weeks and GH-PH-SH at pre (0), 6, 10, and 14 weeks post-separation from GH (see more detail in **Figure 1**).

Significant (p < 0.05) values were shown between groups. We have compared all groups and cell subsets and only significant values for any group combination were shown.

#### Role of Total, Proliferating T Cells, Activating T Cells, Naïve (CD28+CD95−) T Cells, Central Memory (CM, CD28+CD95+) CD4 T Cells, Effector Memory (EM, CD28-CD95+) T Cells, Regulatory (CD25+) T and B Cells, Early Activating (CD69+) T and B Cells, Trafficking (CD62L+) T And B Cells, and Late Activating (HLADR+) T Cells

There were no group or group x time effects on mean values of any other T or B cell markers (including total CD4, total CD8, total CD20, proliferating T cells, activating CD38+ T cells, naïve T cells, central memory CD4 cells, effector memory T cells, regulatory T and B cells, early activating T and B cells, late activating T cells, and trafficking T and B cells), showing no evidence of differences in the effect of removal to single housing vs pair housing on total CD4 and CD8 (**Supplemental Figures S2**-**S4**).

#### Role of Central Memory (CD28+CD95+) CD8 T Cells, Activating (CD38+) and Proliferating (Ki67+) B Cells

A significant treatment group difference in CD8+ central memory (CD28+CD95+) marker expression was detected (**Figure 2A**). Mean frequency of CD8+CM+ T cells following pair separation to single housing was higher compared to group separation to pair housing at all post-separation time points. This same pattern was detected for frequency of activating (CD38+) and proliferating (Ki67+) B cells (**Figures 2B, C**). However, the *groups* mean frequency for those markers did not change over time, providing no evidence supporting the hypothesis.

### Effect of Housing Changes in Peripheral Blood T and B Cell Population Across All Treatment Groups at the Time of Removal From Group Housing and 10 Weeks Post-Separation (Hypothesis 2)

**Figure 3:**
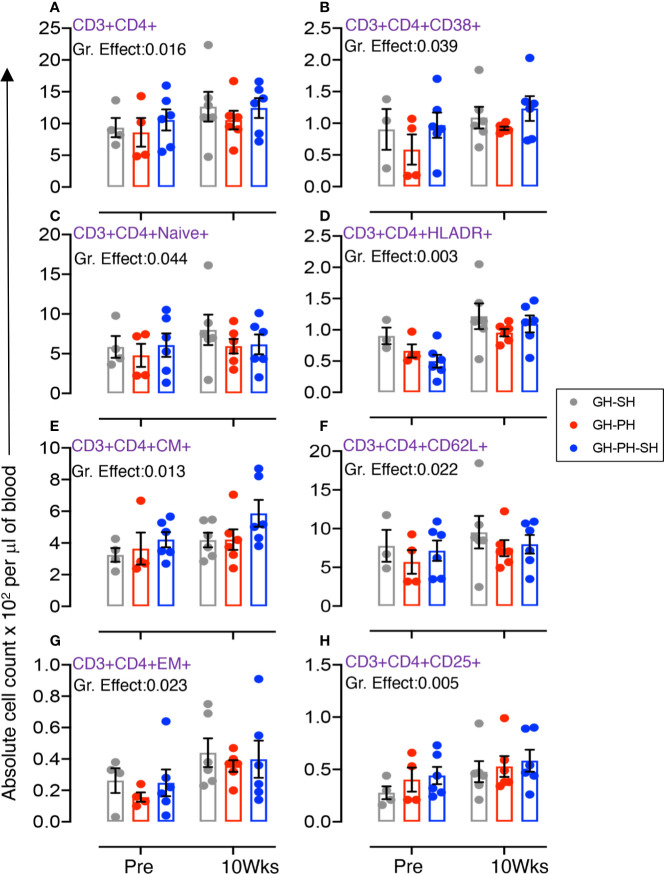
Absolute count of peripheral blood CD4+ T cells and its subpopulation were shown for GH-SH, GH-PH and GH-PH-SH groups. A significant difference in total CD4+ **(A)**, activating CD4+CD38+ **(B)**, CD4+naïve+ (CD28+CD95−) **(C)**, late activating CD4+HLADR+ **(D)**, CD4+central memory (CM) (CD4+CD28+CD95+) **(E)**, trafficking CD4+CD62L+ **(F)**, CD4+effector memory (EM) (CD4+CD28−CD95+) **(G)**, and regulatory CD4+CD25+ **(H)** cells was detected among groups across housing treatment time points. The X axis denotes pre- and post-separation housing time points and the Y axis denotes absolute counts. Individual values from each animal are shown as colored circles with bars showing mean ± standard error for each group.

Eighteen animals assigned to three groups (GH-PH, GH-SH, and GH-PH-SH) were used for these analyses (**Table 1**, **Figure 3**, **Supplemental Figure S5**, and **Supplemental Tables S2**-**S4**).

#### Role of Total, Activating, Naïve, Late Activating, Central Memory, Trafficking, Effector Memory, and Regulatory CD4 T Cells

The total CD4 was significantly different across treatment groups (**Figure 3A**). Similarly, significant differences in mean values of activating, naïve, HLADR+, CM, CD62L+, EM and CD25+ CD4+ T cells were detected across groups (**Figures 3B–H**). No housing x time interactions were detected. Descriptively, the mean value for each CD4 subpopulation was higher than baseline in all treatment groups. Overall, these results provide no evidence supporting the hypothesis that stress markers in animals 10 weeks following removal from group housing vary between long-term pair housing and single housing, with and without a 2 week buffer in pair housing.

#### Role of Total, Early Activating, Naïve, Trafficking, Central Memory, Activating, and Proliferating CD8 T Cells

**Figure 4:**
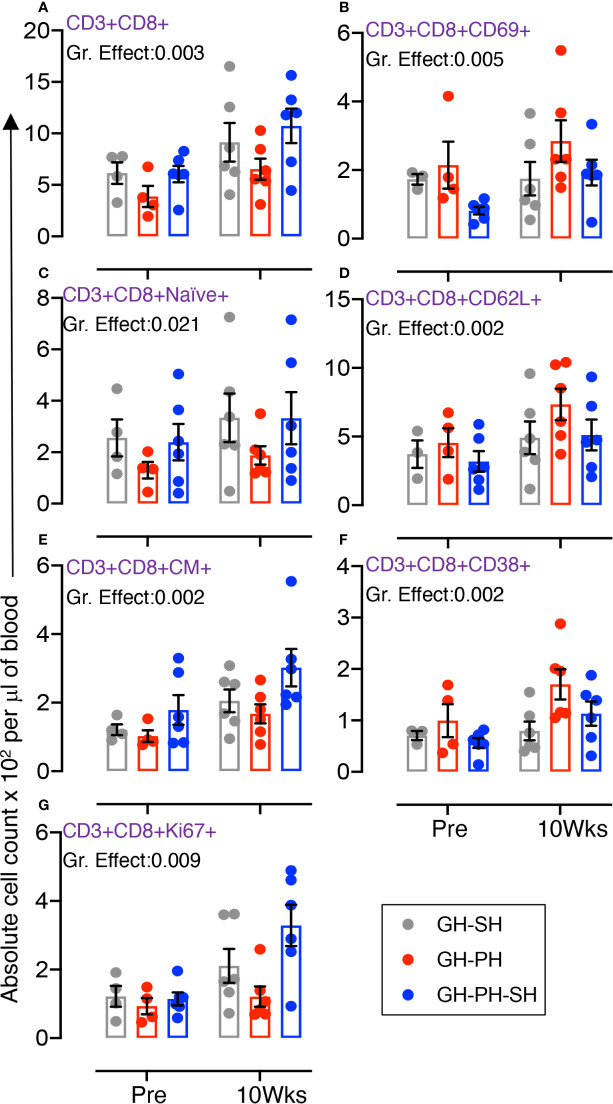
Absolute count of peripheral blood CD8+ T cells and its subpopulation were shown for GH-SH, GH-PH and GH-PH-SH groups. A significant difference in total CD8+ **(A)**, early activating CD8+CD69+ **(B)**, CD8+naïve+ (CD28+CD95−) **(C)**, trafficking CD8+CD62L+ **(D)**, CD8+central memory (CM) (CD8+CD28+CD95+) **(E)**, activating CD8+CD38+ **(F)**, and proliferating CD8+Ki67+ **(G)** cells was detected among groups across housing treatment time points. The X axis denotes pre- and post-separation housing time points and the Y axis denotes absolute counts. Individual values from each animal are shown as colored circles with bars showing mean ± standard error for each group.

The total CD8 was significantly different between groups (**Figure 4A**). Similarly, significant differences in mean values of early activating, naïve, CD62L+, CM, CD38+, and Ki67+ CD8+ T cells were detected between groups (**Figures 4B–G**). No housing x time interactions were detected. Descriptively, the mean value for each CD8 subpopulation was higher than baseline in all treatment groups. Overall, these results provide no evidence supporting the hypothesis that the stress experienced by animals 10 weeks following removal from group housing varies between the use of long-term pair housing and single housing, with and without a 2 weeks buffer in pair housing.

#### Role of Regulating, Proliferating, Activating, and Trafficking CD20+ B Cells

**Figure 5:**
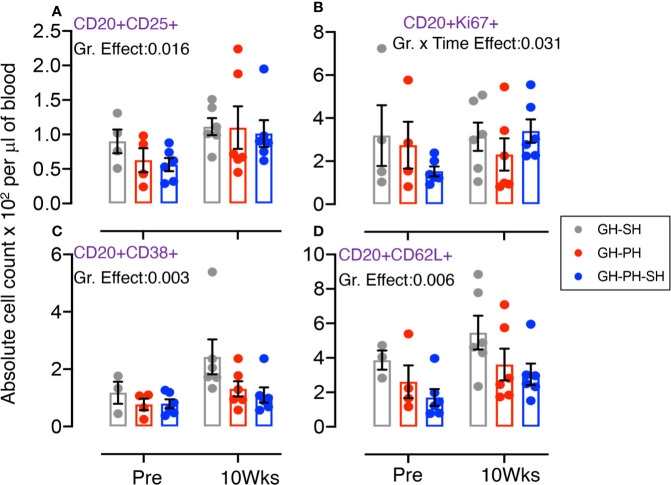
Absolute count of peripheral blood CD20+ B cells and its subpopulation were shown for GH-SH, GH-PH, and GH-PH-SH groups. A significant difference in regulatory CD20+CD25+ **(A)**, proliferating CD20+Ki67+ **(B)**, activating CD20+CD38+ **(C)**, and trafficking CD20+CD62L+ **(D)** cells was detected among groups across housing treatment time points. Interestingly, a significant interaction effect between time and group in proliferation CD8+Ki67+ B cells was detected (p = 0.031). The X axis denotes pre- and post-separation housing time points and the Y axis denotes absolute counts. Individual values from each animal are shown as colored circles with bars showing mean ± standard error for each group.

Significant differences in mean values of CD25+, CD38+, and CD62L+ B cells were detected across groups (**Figures 5A, C, D**). No housing × time interactions were detected. Descriptively, the mean value for each CD8 subpopulation increased following separation from group housing. However, a significant interaction effect between time and treatment group was detected in the frequency of proliferating CD20+ cells (**Figure 5B**). At 10 weeks, levels of proliferating B cells were higher in the GH-PH-SH animals than in the GH-PH group, in which a social partner remains, and the mean proliferating B cells frequency in the GH-PH subjects was lower than the baseline values (**Figure 5B**). This supports the hypothesis that the stress experienced by animals 10 weeks following removal from group housing is lower in pair housed animals than singly housing animals, despite their having had a prior 2 weeks buffer in pair housing.

#### Role of Proliferating CD4, Early Activating CD4, EM CD8, Regulatory CD8, Late Activating CD8, Total B, and Early Activating B Cells

There were no main effects of housing or housing x time interaction effects on the frequency of CD4+Ki67+, CD4+CD69+, CD8+EM+, CD8+CD25+, CD8+HLADR+, total CD20+, and CD20+CD69+ cells (**Supplemental Figure S5**) providing no support *via* the dynamics of those T or B cell populations for the hypothesis that the stress experienced by animals 10 weeks following removal from group housing varies between long-term pair housing and single housing with and without a 2 week buffer in pair housing.

### Effect of Separation From Group to Single Housing Versus the Separation From Pair to Single Housing on Peripheral Blood T and B Cell Population (Hypothesis 3)

**Figure 6:**
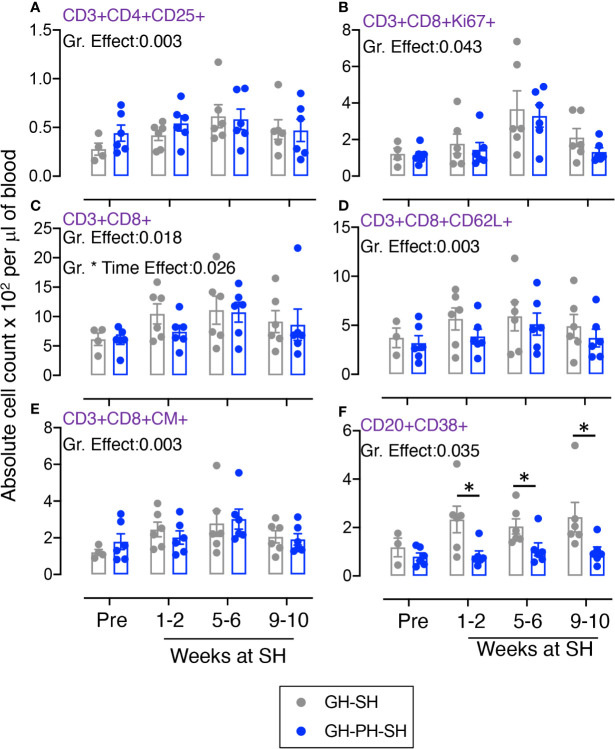
Absolute count of peripheral blood T and B cells and its subpopulation were shown for GH-SH and GH-PH-SH groups. A significant difference in regulatory CD4+CD25+ **(A)**, proliferating CD8+Ki67+ **(B)**, total CD8+ **(C)**, trafficking CD8+CD62L+ **(D)**, central memory (CM) CD8+CD28+CD95+ **(E)**, and activating CD20+CD38+ **(F)** cells was detected between groups across house treatment time points. A significant difference in total CD8+ T cells **(C)** was also detected between GH-SH and GH-PH-SH groups when time and group interaction studied (p = 0.026). The X axis denotes pre- and post-separation housing time points and the Y axis denotes absolute counts. Individual values from each animal are shown as colored circles with bars showing mean ± standard error for each group. Asterisks denote statistical significant differences (p < 0.05) between groups for the specified time points.

Twelve animals assigned to the two groups (GH-SH and GH-PH-SH) were used for these analyses (**Table 1**, **Figure 6**, **Supplemental Tables S2**-**S4**, and **Supplemental Figures S6**, **S7**).

#### Role of Regulating CD4, Proliferating CD8, Total CD8, Trafficking CD8, CM CD8, and Activating CD20+ Cells

Significant differences in mean values of CD4+CD25+, CD8+Ki67+, total CD8+, CD8+CD62L+, CD8+CM+, and CD20+CD38+ cells were detected among treatment groups (**Figures 6A–F**). A significant interaction between time and treatment group was detected for the frequency of total CD8+ T cells (**Figure 6C**). At 2 weeks separation from group to single housing, the frequency of CD8+ T cells were higher in GH-SH subjects compared to one week following the separation from pair into single housing in the GH-PS-SH subjects. However, this pattern was not observed as the tenure of single housing in both groups increased. These data suggest that housing change has a significant impact on this early increase in CD8 population in GH-SH compared to GH-PH-SH group (**Figure 6C**). We have also observed a significant difference in the activated CD20 population between GH-SH and GH-PH-SH groups. At baseline, the B cell activation marker was not significantly different. But at 1-2, 5-6, and 9-10 weeks at their single housing condition, a significantly higher expression of activating B cells were detected in GH-SH compared to GH-PH-SH animals (**Figure 6F**). This finding, while not supporting the hypothesis regarding the relative degree of stress associated with separation to group housing versus pair to single housing, does support the idea that the brief period of pair housing may provide a buffer to singly housed animals.

#### Role of Total CD4 and CD20, Activating CD4 and CD8, Naïve CD4 and CD8, CM CD4, EM CD4 and CD8, Proliferating CD4 and CD20, Regulating CD4 and CD20, Early Activating CD4, CD8, and CD20, Late Activating CD4 and CD8, and Trafficking CD4 and CD20 Cells

There were no main or interaction effects between time and treatment groups on the frequency of total CD4, CD4+CD38+, CD4+naïve+, CD4+CD69+, CD4+CM+, CD4+HLADR+, CD4+EM+, CD4+CD62L+, CD4+Ki67+, CD4+CD25+, CD8+naïve+, CD8+EM+, CD8+CD69+, CD8+HLADR+, CD8+CD38+, total CD20+, CD20+CD25+, CD20+Ki67+, CD20+CD62L+, and CD20+CD69+ cells (**Supplemental Figures S6**, **S7**) providing no evidence that there is a difference in stress experienced in the separation from group to single housing versus pair to single housing.

### Dynamics of CDMV-Specific T Cell Memory Responses

**Figure 7:**
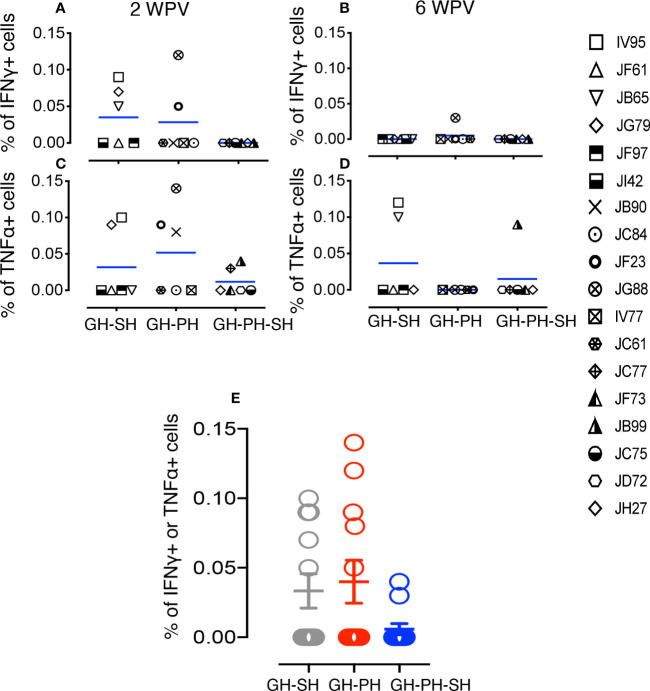
Measles and canine distemper specific CD4 T cell responses in canine distemper and measles vaccinated macaques. Intracellular IFNγ responses **(A, B)** and TNFα responses **(C, D)** were measured against lysed Canine Distemper measles vaccine (CDMV) at 2 weeks and 6 weeks post-vaccination (WPV) in three different housing groups of macaques. CDMV specific IFNγ or TNFα responses in CD4+ T cells were shown for all three groups at 2 WPV with mean ± standard error **(E)**. Blue lines in **(A–D)** graphs represent group means for each group. Criteria for a positive cytokine response was a two-fold increase in frequency for measles and canine distemper antigen and cytokine above the medium control culture. All values were subtracted from medium control before analysis.

**Figure 8:**
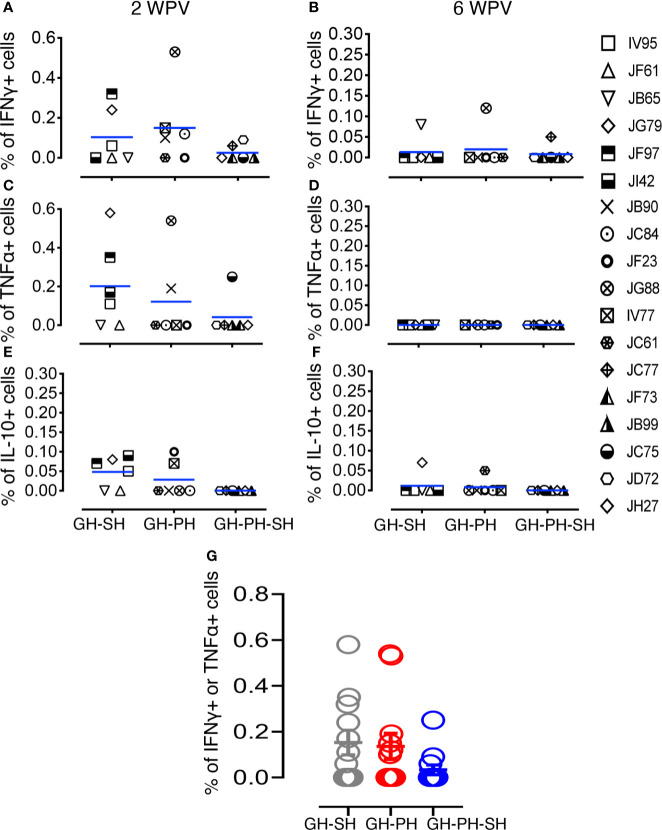
Measles and canine distemper specific CD8 T cell responses in canine distemper and measles vaccinated macaques. Intracellular IFNγ responses **(A, B)**, TNFα responses **(C, D)** and IL-10 responses **(E, F)** were measured against lysed Canine Distemper measles vaccine (CDMV) at 2 weeks and 6 weeks post-vaccination (WPV) in three different housing groups of macaques. CDMV specific IFNγ or TNFα responses in CD8+ T cells were shown for all three groups at 2 WPV with mean ± standard error **(G)**. Blue lines in **(A–F)** graphs represent group means for each group. Criteria for a positive cytokine response was a two-fold increase in frequency for measles and canine distemper antigen and cytokine above the medium control culture. All values were subtracted from medium control before analysis.

We investigated the importance of housing configuration on CDMV specific cytokine responses in all CDMV vaccinated rhesus macaques (**Figures 7**, **8**). Representative CFC results for IFNγ and TNFα cytokine production by CD8+ T cells are shown from a CDMV vaccinated rhesus macaque (**Supplemental Figure S1**). No significant main effects of housing or housing x time interaction effects were detected. One subject from the GH-SH and one subject GH-PH-SH groups had no detectable antigen-specific cytokine responses throughout the course of this study (**Figures 7**, **8**). Overall, positive cytokine responses were detected in 21 out of 23 rhesus macaques for either IFNγ, TNFα, or IL-10 cytokines in CD4 and/or CD8+ T cells at some time point. Descriptively, consistently increased cytokine responses were detected at 2 weeks post-vaccination (wpv) compared to 6 wpv in CD4 and CD8+ T cells. The percentage of TNFα and IFNγ responses ranged from 0.00% to 0.58% and 0.00% to 0.53%, respectively, and an increased cytokine response was detected in the CD8 T cell population. Descriptively, animals from the GH-PH-SH group had lower cytokine responses than those in the other treatment groups. The mean value of CDMV specific TNFα and IFNγ responses were not statistically significant within treatment groups over time for either CD4 or CD8 T cells (**Figures 7**, **8**). IL-10 was also measured in all treatment groups. No main or interaction effects on the mean frequency of IL-10 responses were detected (**Figure 8**). The majority of the animals from the GH-SH group had increased antigen specific IL-10 responses detected in CD8+ T cells at 2 wpv. Descriptively, the IL-10 specific responses diminished by 6 wpv in the GH-SH group. Interestingly, GH-PH-SH animals had low to undetectable IL-10 responses at 2 and 6 wpv time points (**Figure 8**). Overall, we have detected fewer vaccine specific cytokine producing cells in the GH-PH-SH group compared to other groups post-vaccination. However, we found no significant effect supporting a relationship between vaccine-specific immune responses and housing condition or history.

### Measles Neutralization Antibody Titers Following Vaccination

**Figure 9:**
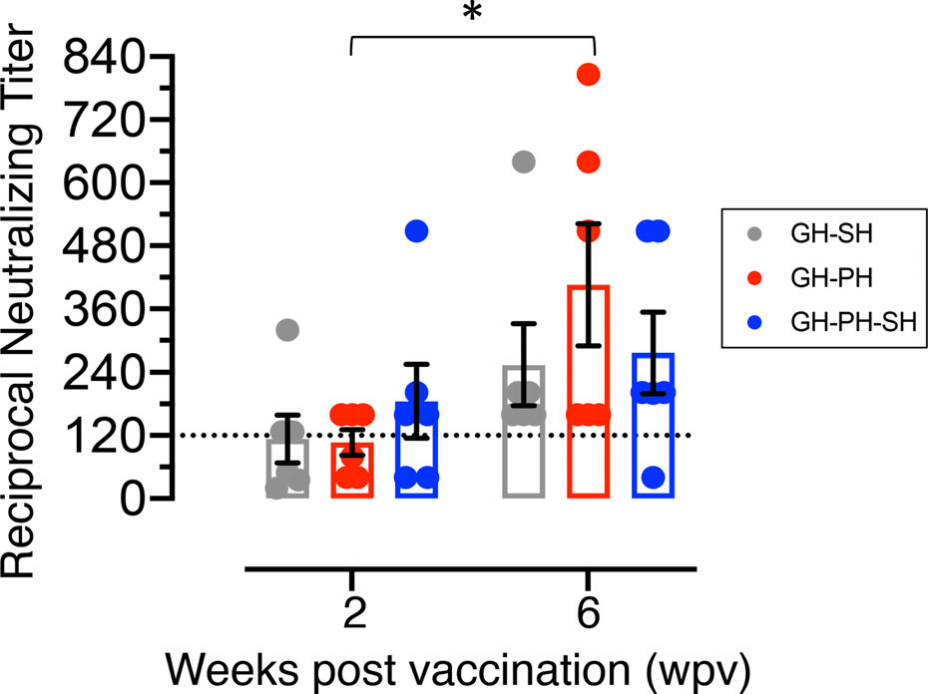
Mean neutralizing antibody titers (mean ± standard error) of rhesus macaques vaccinated with canine distemper and measles vaccine were shown for GH-SH, GH-PH and GH-PH-SH groups. GH-PH animals demonstrated the highest neutralizing antibody response compared to the other groups at 6 weeks post vaccination (wpv). In contrast, GH-SH animals generated the lowest neutralizing antibody titers at 6 wpv, however the differences in responses were not statistically significant. Note, one animal from GH-PH-SH group had lower level of protective immunity (neutralizing titers 40) at 6 wpv time point compared to any group tested. Statistically significant differences were shown between pre and post vaccination timepoints in different housing groups (* represent P<0.05). The dotted line in the Y axis represents the accepted protective titer in humans (120).

Serum neutralization antibody (NAb) titers to measles virus were analyzed at 2 and 6 wpv in all animals (**Figure 9**). No significant differences due to the effects of housing or housing x time interaction were detected between groups. JC77 from GH-PH-SH group had no protective neutralizing measles titer (neutralizing titer 40) at 6 wpv, based on the accepted protective neutralizing antibody titer of 120 or above for humans (26) (**Figure 9**). Thus, we found no support for the hypothesis that housing or housing changes influence vaccine-specific immune response.

## Discussion

The TNPRC maintains a large rhesus monkey breeding colony for use in biomedical research. Animals in the breeding colony are housed in large, outdoor social groups from which individuals are removed for assignment to research projects. Animals may be transitioned to single housing, but if more than one individual is removed from the same group, the TNPRC pursues a practice of moving the animals as familiar pairs into indoor caging as a method to decrease potential separation distress. Some pairs may remain together long-term, while others may subsequently be separated into single housing depending on research needs. These practices provided an opportunity to contribute to the literature on the relationship between social housing, changes in social housing, stress, and downstream physiological effects.

Acceptance of social housing as the gold standard for maintaining NHPs in a research setting is arguably one of the greatest strides made to improve psychological wellbeing since the advent of the use of these species in biomedical research. There is strong justification for social housing of NHPs as the lack of same-species contact has been associated with increased self-injurious behavior and stereotypies (or species-inappropriate repetitive behaviors) (27). Beyond the psychosocial implications, these behaviors also have the potential to interfere with research outcomes by themselves, possibly altering immune function or truncating study timelines for humane euthanasia of animals with severe self-injurious behavior.

Potential changes in immune response influenced by social housing status and stability introduce the possibility of confounds to research (28). At the same time, it is to the benefit of the quality of research and reproducibility if study subjects are physiologically and behaviorally normal and stable over the duration of research project timelines. This can best be supported with social housing, as it can reduce the chronic stress of social isolation as well as buffer animals from stressors that would otherwise be more likely to create research confounds (8, 10, 11, 29, 30).

Where potential confounds may be induced, it is valuable to the field to be aware of their existence, as well as their magnitude and the timing of effects. Housing requirements may change over the course of a study for a variety of reasons. The housing change may be planned (e.g., animals separated for viral challenge until confirmation of infectious status, or unplanned separations, due to the development of incompatibility in socially housed animals, changing availability of social partners, and other practical limitations) (31). This paper also illustrates the value of detailing the social housing status of NHP subjects in the description of methods in research publications for comparing the findings of different studies and potentially enhancing reproducibility.

This study was subject to several limitations. First, the sample size of subjects was modest. Second, by necessity the study design introduced confounds. All animals were moved from outdoor to indoor housing in addition to being separated from their large social groups. Placement in caging and indoor housing was no doubt a significant stressor. Also, the GH-PH-SH treatment group underwent a longer period of tenure in caging, necessitated in order to compare the separation from group to pair housing with the separation from pair to single housing.

Third, management limitations resulted in the housing timeline not being ideal for addressing all hypotheses. For instance, data were available from a timepoint 2 weeks after removal from group housing into pair housing, but only one week after separation from pair to single housing. This discrepancy could have affected the results relating to hypothesis 2 (The separation from group to single housing will result in greater levels of stress than the separation from pair to single housing). It is possible that the elapsed time from separation to blood sampling played a role in the elevated frequency of CD8+ T cells after separation into single housing compared to separation from group to pair housing. An additional discrepancy pertained to the timing of the measles vaccine relative to social separation; two treatment groups received their vaccine 4 weeks after separation from group housing, but the treatment group whose last social change prior to inoculation was the separation of pairs into single housing received the vaccine 3 weeks after separation. This discrepancy could have resulted in a weakened vaccine-specific immune response in the GH-PH-SH subjects. Nonetheless, we did not see significantly weaker immune response in GH-PH-SH animals, suggesting that the discrepancy did not confound the study.

Fourth, data were not available for all animals and all time-points, most notably, immediately prior to separation from pair housing to single housing, which would have provided for a tighter comparison of the effects of group-to-pair separations and pair-to-single housing separations. Lastly, the lack of monitoring vaccine induced responses for an extended period of time following separation which might have confirmed the dynamics of T and B cell proliferation, activation, and their function.

On the other hand, several strengths of this study should be noted. First, the size and age/sex composition of the groups from which subjects were drawn were closely matched. All subjects had exactly the same rearing background and prior social experience. Once in indoor housing, all subjects were housed in one room of unvarying room composition and arrangement, introducing no potential confounding social stressors or effects of room position on stress (32, 33). Levels of macroenvironmental disruption (e.g., human traffic and activity), which can influence heart rate in rhesus macaques (34) were held steady over the course of the study. Outdoor macroenvironmental effects that could influence stress levels, including weather, enclosure repairs, and changes in the composition of social groups, were also not sources of potential confounds. It would not have been possible to control for macroenvironmental effects if the study were conducted outdoors.

This longitudinal study monitored stress-induced changes in the immune system. We did not find support for our first hypothesis as we did not see any significant difference in any of the T and B cell immune responses depending on whether group-housed subjects were moved into pair housing or single housing over our 10 weeks post-separation observation period.

In our second hypothesis, our study demonstrated significant differences in proliferating (Ki67) B cell markers across the GH-SH, GH-PH, and GH-PH-SH subjects 10 weeks after removal from group housing. Nuclear protein Ki67 is expressed only in proliferating cells and its level increases from the G1 phase to mitosis and is also considered a novel marker to measure antigen specific T cell proliferation *in vitro* (35, 36). Increased proliferation in GH-PH-SH animals compared to GH-SH groups at 10 weeks after removal from group housing suggests that the second separation that occurred in the GH-PH-SH group increased levels of B cell proliferation compared to the single separation that happens in GH-SH animals. Whereas GH-PH group had a detectable lower frequency of proliferating B cells at 10 weeks post-separation compared to the other groups suggesting that paired housing causes less stress to B cell proliferation, but that brief pair housing may not mitigate the effects of long-term single housing.

In our third hypothesis, we have detected a significant difference in singly housed subjects’ CD8 population between GH-SH and GH-PH-SH groups following separation from group versus separation from pair housing, such that the separation from group housing to single housing in GH-SH caused greater CD8 T cell expansion than separation from pair to single housing. The CD8 T cell expansion is also evident by the increased expression of a trafficking molecule in the GH-SH group compared to the GH-PH-SH animals, despite both treatment groups being housed singly at the time of sample collection. A study by Mays et al. supports our finding where repeated social disruption stress augments clonal expansion of CD8+ T cells during primary influenza infection (37). This finding supports the use of short-term pair housing to buffer individuals from the transition from outdoor group housing to indoor single housing. We did not observe any changes in total CD4 and CD20 populations, suggesting that housing changes have less impact on these cell types. We have not detected any other significant differences in T and B cell phenotypic parameters between groups over time. However, we have observed several group effects between the three treatment groups which were not dependent on the time point. Significantly more activated B (CD20+CD38+) cells were detected in GH-SH animals compared to GH-PH-SH animals, both at the time point involving single housing. However, we did not find any correlation between activated B cell population and measles specific NAb titers among animals.

Because antibody response to an immune challenge is frequently used as a measure of general immune function *in vivo* for both immunological and psychosocial research in humans (38), we sought to elucidate how changes in social housing might impact different T and B cell activation, proliferation and other phenotypic markers, and immune response to vaccination with a commercially available CDMV, which is given routinely as part of a preventive health program. An appropriate measles vaccine mediated NAb titer is critical for providing protective immunity for the collective health of the colony. In addition to protective NAb responses, measles vaccine has also been shown to generate cytotoxic T cell responses in rhesus macaques (39–41). Routine measles vaccination administered as part of a preventive medicine program allowed us to study the vaccine mediated immune response in animals, which might provide valuable information about the importance of NHP housing status in future research studies using a variety of vaccine candidates.

In general, both animals and humans respond to stress through stimulation of the sympathetic nervous system and the hypothalamic-pituitary-adrenal axis, which ultimately results in enhanced glucocorticoid secretion. These steroid hormones have a direct effect on immune function *via* binding to various populations of immunocompetent cells (42). One such effect is elicited by way of inhibition of proinflammatory cytokine (TNFα) production, by which glucocorticoids directly affect adaptive immunity by shifting T-helper-1 (Th1) to T-helper-2 (Th2) cells (43). The most robust vaccine specific T cell responses were detected in the treatment group involving long-term pair housing, 6 weeks after removal from group housing. Also, the majority of these animals had low IFNγ and TNFα responses compared to other groups. This suggests that stress induced by the separation from partners has a major impact on the reduction in vaccine mediated Th1 responses compared to other groups examined. This observation mirrors a recent finding regarding cold stress where mice housed at lower temperature had decreased antigen-specific T cell responses to a live vaccine compared to mice housed at thermoneutral temperatures (44). IL-10, an immunomodulatory cytokine, is an inhibitory factor for Th1 cytokine responses (20, 45). In our study, a majority (4 out of 6) of GH-SH animals were able to produce measles specific IL-10 compared to GH-PH animals (2 out of 6). Our observation contradicts a previous study, in which singly-housed adult male macaques had relatively low LPS stimulated IL-10 production compared to paired-housed animals (8).

Acute elevation of the hormones and neurotransmitters associated with short term stress is physiologically advantageous and allows an organism to adapt and cope with stressful stimuli in close temporal proximity. Indeed, in healthy individuals, a period of immuno-enhancement has been shown to immediately follow short-term stressors, such as the production of a more robust antibody response to vaccination immediately following physical or mental stress (2). The findings of our study, while not statistically significant, are consistent with this concept. Primary CDMV immunization occurred early in the GH-PH-SH group (3 weeks after separation) compared to GH-SH and GH-PH groups (4 weeks after separation). Thus, this housing change stressor prior to primary CDMV immunization might have had a positive impact on increased measles NAb IgG responses in GH-PH-SH group compared to other groups at 2 wpv time point. However, at 6 wpv, which was 9–10 weeks after housing reorganization, the GH-PH group had the highest neutralizing titer of any group suggesting that in the long term, social housing has a positive impact on inducing increased vaccine specific IgG responses compared to single housing. In this study, we have only monitored the plasma NAb titers, which does not predict the protective outcome from pathogenic measles challenge. However, increased titered NAbs are necessary for protection from a pathogenic wild-type measles virus challenge (46). Therefore, our study is consistent with our fourth hypothesis. We have observed descriptively stable and increased T and B cell responses in the GH-PH group compared to the rest of the groups tested.

In conclusion, we have identified two important cellular phenotypic biomarkers (frequency of CD8 T cells and proliferating B cells) in adolescent male rhesus macaques that reflect an effect of housing status on experimental outcomes. Our results are consistent with the idea that singly housed individuals experience more stress than pair housed animals, and suggest that it may be most important to account for the effects of separation from group to single housing than from pair to single housing when assessing potential confounding effects of housing on experiments. Based upon our findings, we recommend a short period of pair housing to buffer animals from removal from group housing even when they must ultimately be housed in single housing for experimental purposes. We were unable to detect any significant differences in measles specific T or B cell responses between groups in our study, suggesting that research evaluating vaccine response may not be confounded by housing treatment. However, one of the GH-PH-SH animals had the lowest NAb titer against vaccine at 6 wpv time point compared to other groups examined. Future studies involving additional animals and a longer time-span could better detect whether our observations hint at a larger pattern that our study did not detect, and enable us to better understand the long-term effect of housing separation on immune activation and vaccine specific responses. Finally, our study suggests that paired housing is beneficial to research and welfare.

## Data Availability Statement

All datasets presented in this study are included in the article/**Supplementary Material**.

## Ethics Statement

The animal study was reviewed and approved by IACUC Committee, Tulane National Primate Research Center.

## Author Contributions

The overall planning, direction, and design of the experiment were carried out by BP, KB, and RB. BP, KB, AJ, PA, KR-L, JB, and RB carried out animal scheduling, sample processing and other experiments. BP designed the flow cytometry panels. BP and AJ analyzed the flow data. SS performed the statistical analysis. BP wrote the manuscript with input from all authors. All authors contributed to the article and approved the submitted version.

## Funding

The study was supported by National Institutes of Health grants R01DK109883, P51OD011104, U42OD024282, and U42OD010568.

## Conflict of Interest

The authors declare that the research was conducted in the absence of any commercial or financial relationships that could be construed as a potential conflict of interest.

## Supplementary Material

Supplementary Figure 1 Intracellular cytokine flow cytometry for IFNγ and TNFα responses from a representative measles and canine distemper vaccinated macaque. Cells were gated first on singlets, lymphocytes, followed by live cells and then on CD3+ T cells and subsequently on CD3+CD4+ and CD3+CD8+ T cell subsets. The percentages of total IFNγ and TNFα+CD8+ T cells are shown in each box of the plots. Note that this macaque had detectable Canine Distemper measles vaccine (CDMV) specific IFNγ and TNFα responses at 2 weeks post-CDMV vaccination.

Supplementary Figure 2 Absolute count of peripheral blood CD4+, proliferating CD4+Ki67+, CD4+naïve+ (CD28+CD95−), activating CD4+CD38+, CD4+central memory+ (CM, CD28+CD95+), trafficking CD4+CD62L+, CD4+effectory memory+ (EM, CD28-CD95+), early activating CD4+CD69+, regulating CD4+CD25+, and late activating CD4+HLADR+ T cells were shown for GH-SH and GH-PH groups. No significant main or interaction effects between treatment group and time were detected for the above T cell population. The X axis denotes pre- and post-separation housing time points and the Y axis denotes absolute counts. Individual values from each animal are shown as colored circles with bars showing mean ± standard error for each group.

Supplementary Figure 3 Absolute count of peripheral blood CD8+, early activating CD8+CD69+, CD8+naïve+ (CD28+CD95+), trafficking CD8+CD62L+, CD8+ effector memory (EM, CD28-CD95+), late activating CD8+HLADR+, regulating CD8+CD25+, activating CD8+CD38+, and proliferating CD8+Ki67+ T cells were shown for GH-SH and GH-PH groups. No significant main or interaction effects between treatment group and time were detected for the above T cell population. The X axis denotes pre- and post-separation housing time points and the Y axis denotes absolute counts. Individual values from each animal are shown as colored circles with bars showing mean ± standard error for each group.

Supplementary Figure 4 Absolute count of peripheral blood CD20+, trafficking CD20+CD62L+, activating/regulating CD20+CD25+, and early activating CD20+CD69+ B cells were shown for GH-SH and GH-PH groups. No significant main or interaction effects between treatment group and time were detected for the above B cell population. The X axis denotes pre- and post-separation housing time points and the Y axis denotes absolute counts. Individual values from each animal are shown as colored circles with bars showing mean ± standard error for each group.

Supplementary Figure 5 Absolute count of peripheral blood proliferating CD4+Ki67+, early activating CD4+CD69+, CD8+effector memory (EM, CD28-CD95+), regulatory CD8+CD25+, late activating CD8+HLADR+, total CD20+, and early activating CD20+CD69+ cells were shown for GH-SH, GH-PH and GH-PH-SH groups. No significant main or interaction effects between treatment group and time were detected for the above T or B cell population. The X axis denotes pre- and post-separation housing time points and the Y axis denotes absolute counts. Individual values from each animal are shown as colored circles with bars showing mean ± standard error for each group.

Supplementary Figure 6 Absolute count of peripheral blood CD4+, activating CD4+CD38+, CD4+naïve+ (CD28+CD95+), early activating CD4+CD69+, CD4+central memory+ (CM, CD28+CD95+), late activating CD4+HLADR+, CD4+ effector memory (EM, CD28-CD95+), trafficking CD8+CD62L+, proliferating CD4+Ki67+, and regulatory CD4+CD25+ T cells were shown for GH-SH and GH-PH-SH groups. No significant main or interaction effects between treatment group and time were detected for the above T cell population. The X axis denotes pre- and post-separation housing time points and the Y axis denotes absolute counts. Individual values from each animal are shown as colored circles with bars showing mean ± standard error for each group.

Supplementary Figure 7 Absolute count of peripheral blood CD8+naïve+ (CD28+CD95+), total CD20+, CD8+effector memory+ (EM, CD28-CD95+), regulating/activating CD20+CD25+, early activating CD8+CD69+, proliferating CD20+Ki67+, late activating CD8+HLADR+, trafficking CD20+CD62L+, activating CD8+CD38+, and early activating CD20+CD69+ cells were shown for GH-SH and GH-PH-SH groups. No significant main or interaction effects between treatment group and time were detected for the above T and B cell population. The X axis denotes pre- and post-separation housing time points and the Y axis denotes absolute counts. Individual values from each animal are shown as colored circles with bars showing mean ± standard error for each group.
