# Supplementary figures and images for: Effects of Social Housing Changes on Immunity and Vaccine-Specific Immune Responses in Adolescent Male Rhesus Macaques
Source: Front Immunol. 2020 Oct 15;11:565746. doi: 10.3389/fimmu.2020.565746 (PMC7593645; doi:10.3389/fimmu.2020.565746)

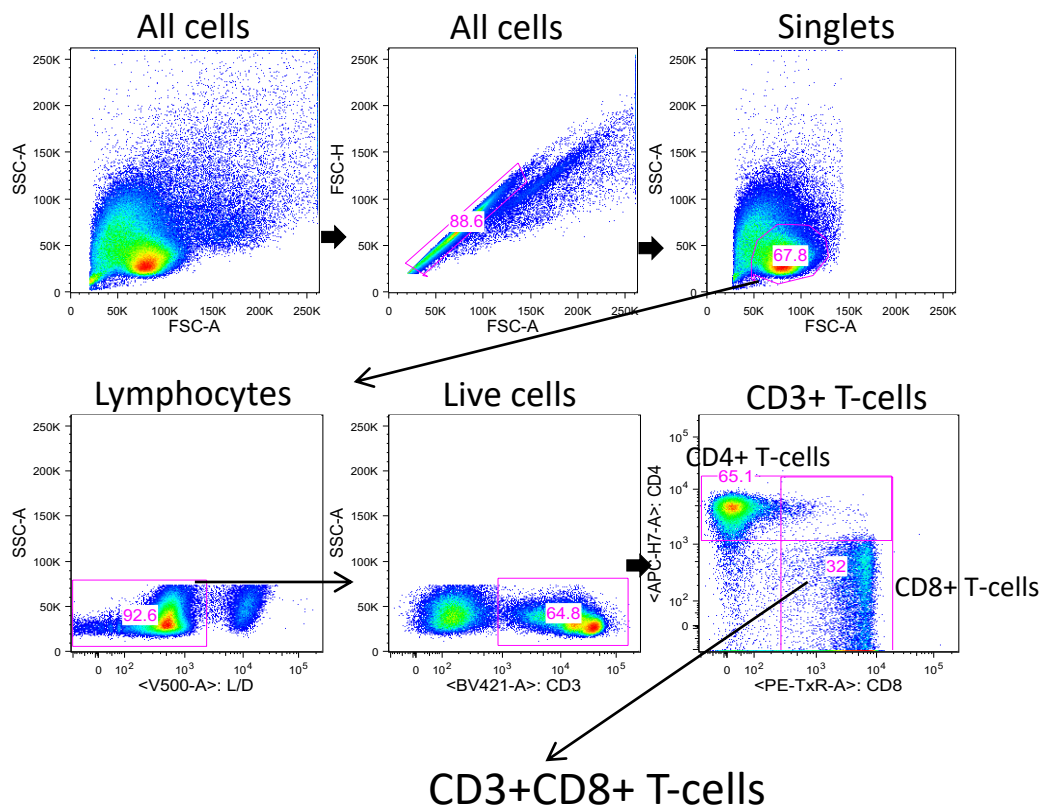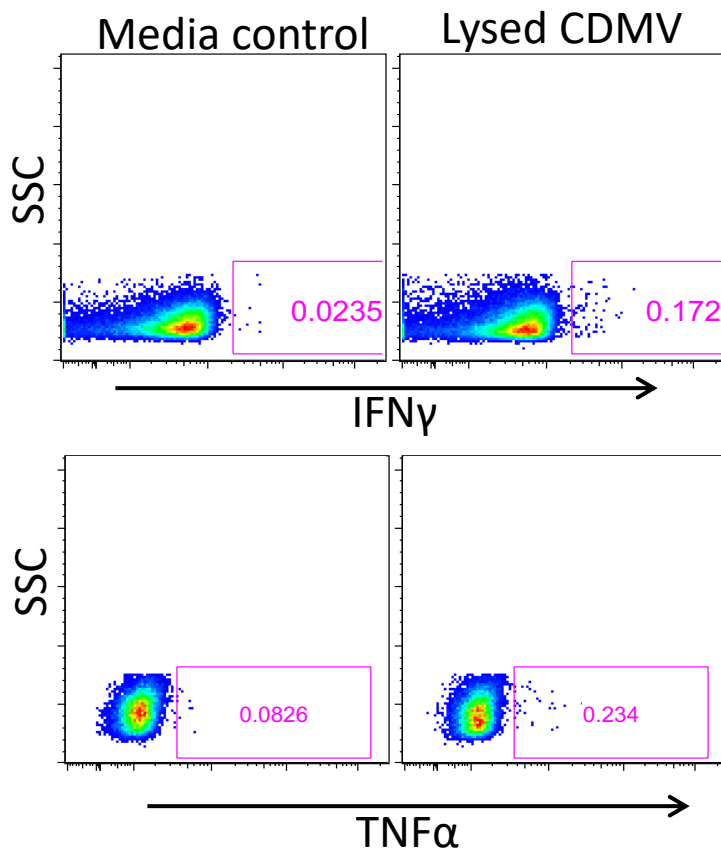

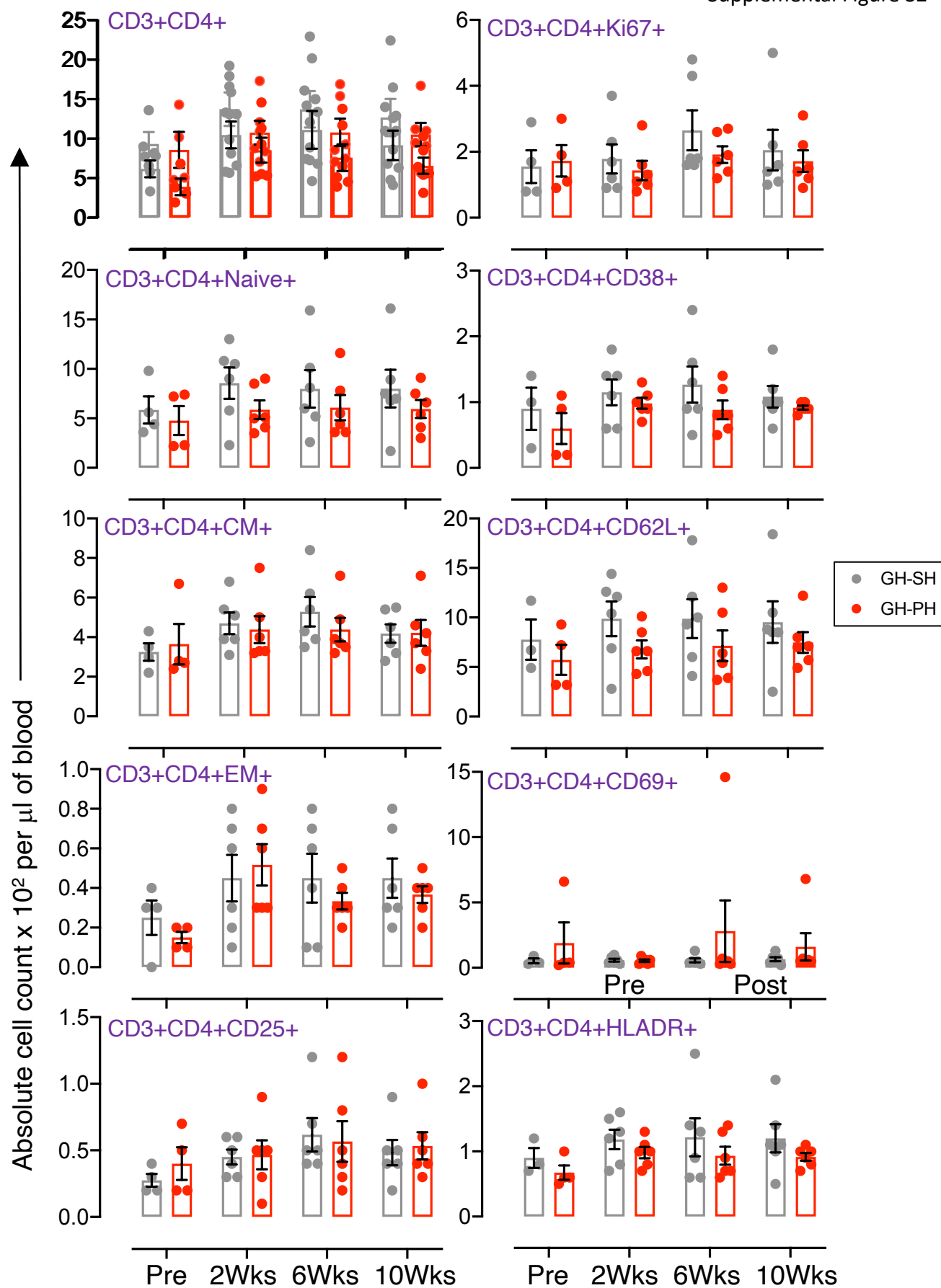

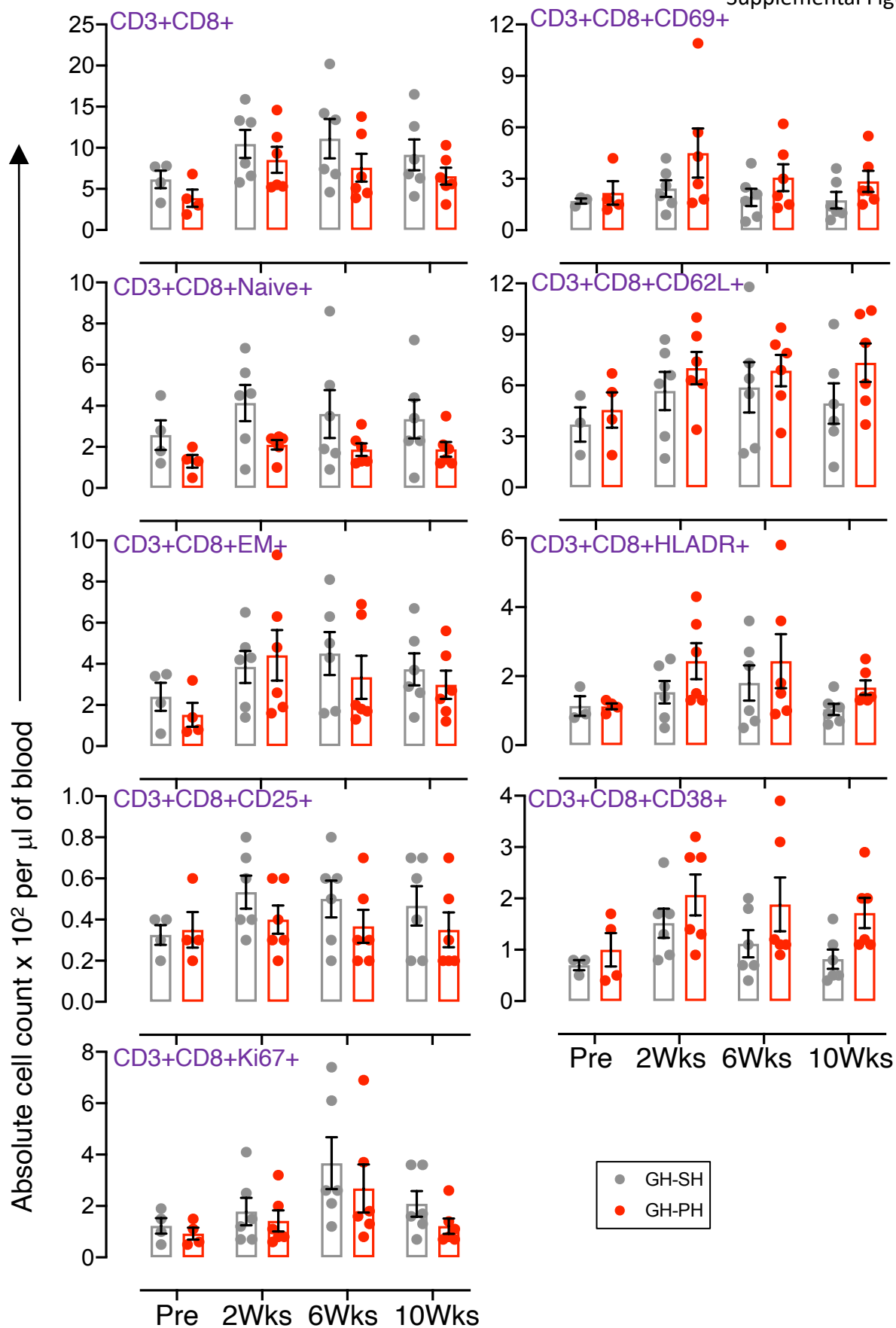

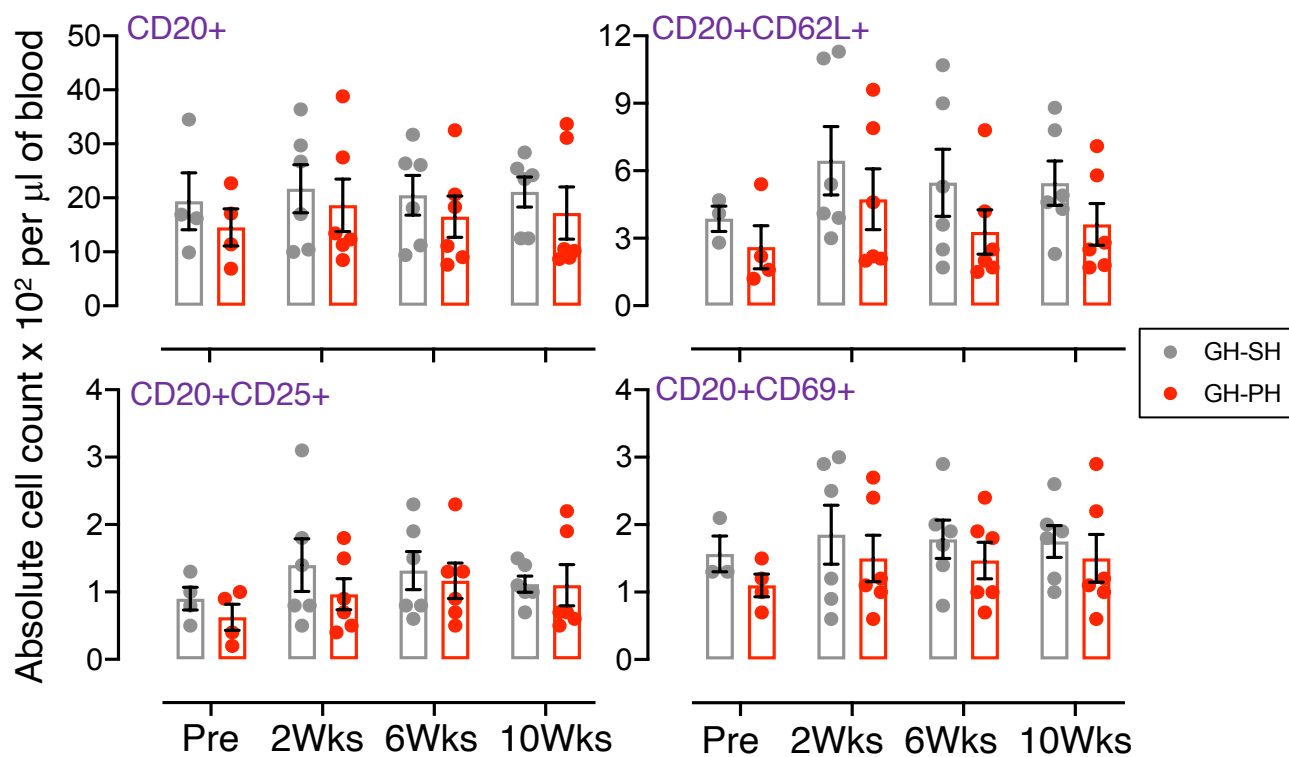

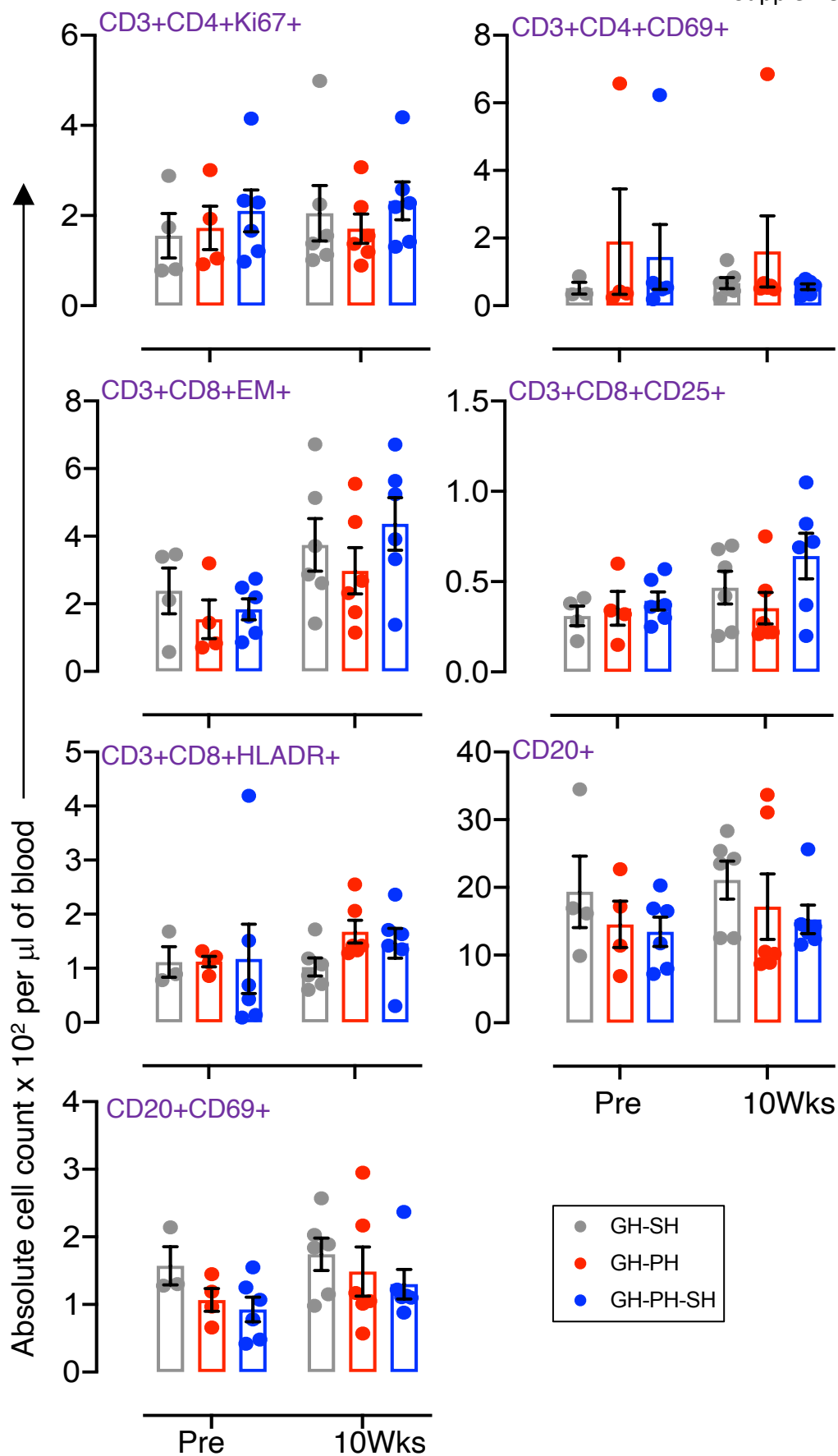

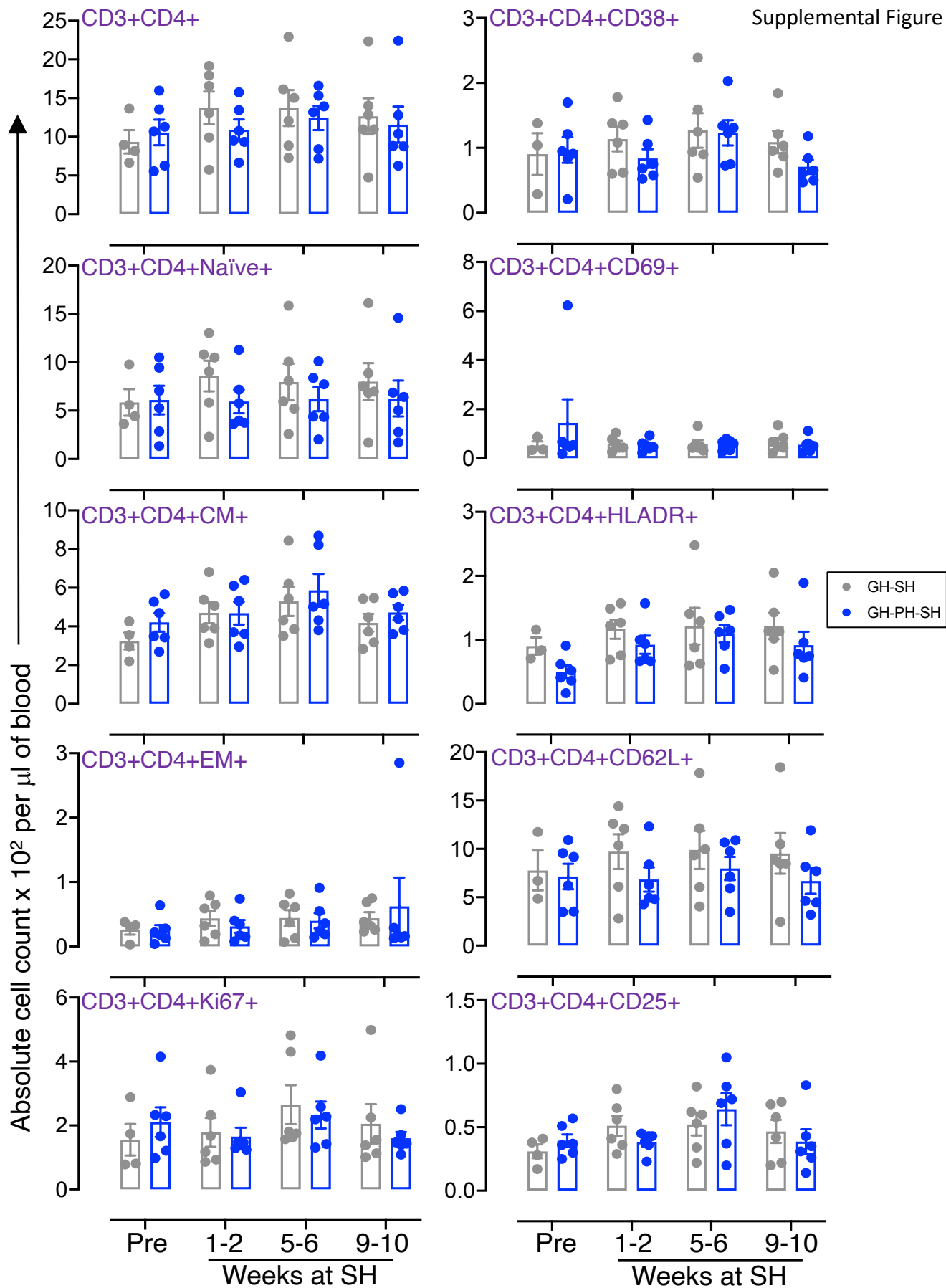

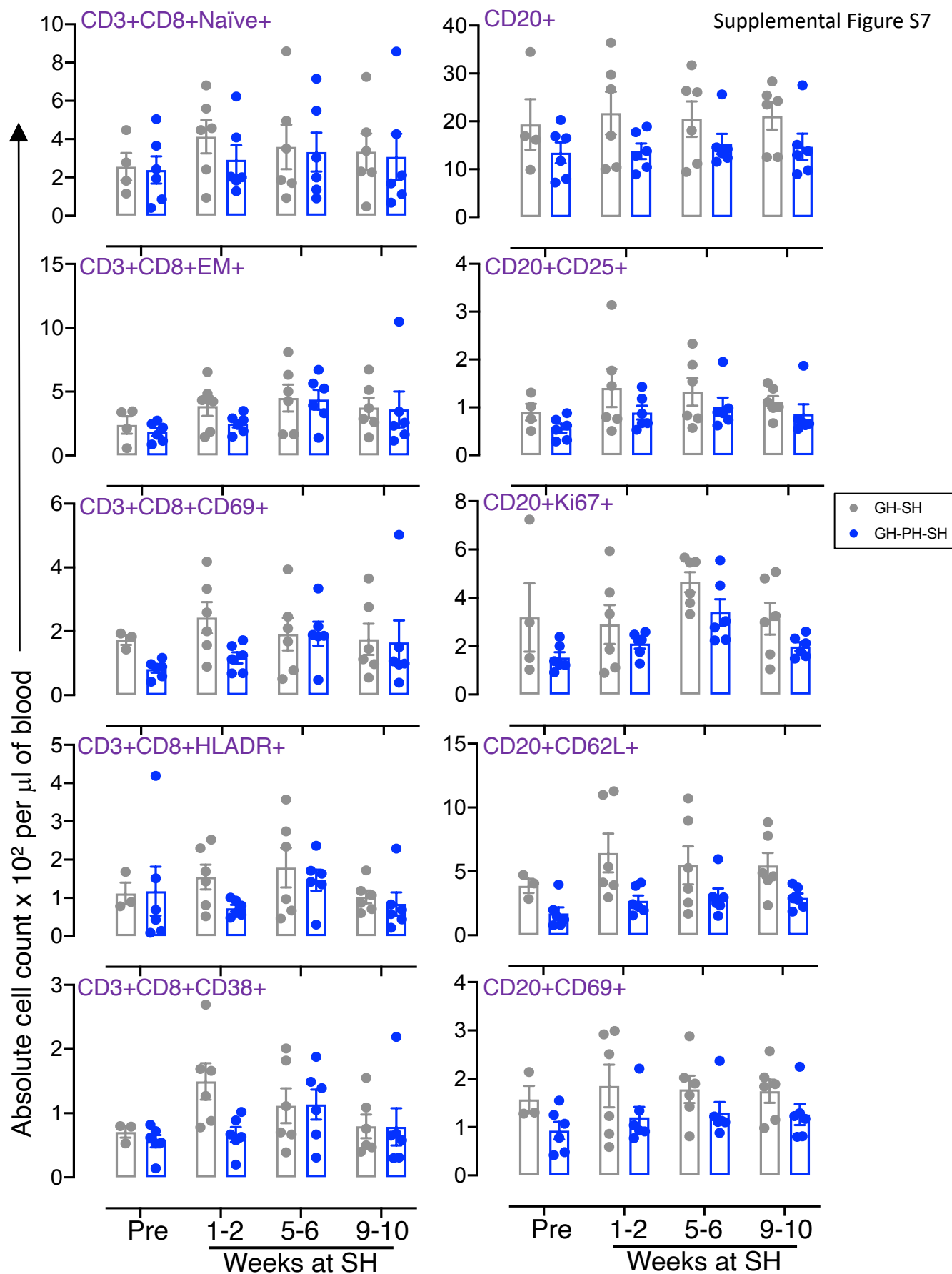

Supplement: Supplementary Figure 1 — Intracellular cytokine flow cytometry for IFNγ and TNFα responses from a representative measles and canine distemper vaccinated macaque. Cells were gated first on singlets, lymphocytes, followed by live cells and then on CD3+ T cells and subsequently on CD3+CD4+ and CD3+CD8+ T cell subsets. The percentages of total IFNγ and TNFα+CD8+ T cells are shown in each box of the plots. Note that this macaque had detectable Canine Distemper measles vaccine (CDMV) specific IFNγ and TNFα responses at 2 weeks post-CDMV vaccination. [file DataSheet_1.pdf]
