# Supplementary material for: Effects of Social Housing Changes on Immunity and Vaccine-Specific Immune Responses in Adolescent Male Rhesus Macaques
Source: Front Immunol. 2020 Oct 15;11:565746. doi: 10.3389/fimmu.2020.565746 (PMC7593645; doi:10.3389/fimmu.2020.565746)
Supplement: Supplementary file 2 [file DataSheet_2.pdf]

Supplementary Table S1. List of monoclonal antibodies used for T and B-cell phenotyping and antigen specific cytokine flow cytometry assays.

Antibody	Clone	Source
Anti-human CD3	SP34-2	BD Biosciences, CA
Anti-human CD8	SK1	BD Biosciences, CA
Anti-human CD20	B9E9	Beckman Coulter, CA
Anti-human CD25	M-A251	Biolegend, CA
Anti-human CD28	CD28.2	BD Biosciences, CA
Anti-human CD38	OKT10	NHP Reagent Resource Program, NIH
Anti-human CD49d	9F10	BD Biosciences, CA
Anti-human CD62L	SK11	BD Biosciences, CA
Anti-human CD69	FN50	Biolegend, CA
Anti-human CD95	DX2	BD Biosciences, CA
Anti-human HLA-DR	G46-6	BD Biosciences, CA
Anti-human IFNγ	B27	Biolegend, CA
Anti-human IL-10	JES3-9D7	Biolegend, CA
Anti-mouse Ki67	B56	BD Biosciences, CA
Anti-human TNFα	Mab11	Biolegend, CA

Supplemental Table S2: Mean ± Standard Error (SE) and Confidence Interval (CI) of different subsets of CD4+ T cells at different housing groups*

Time points In Weeks	Cell Subsets	GH-SH		GH-PH		GH-PH-SH	
		**Mean ± SE**	**Cl (95%)**	**Mean ± SE**	**Cl (95%)**	**Mean ± SE**	**Cl (95%)**
Pre (0)	CD3+CD4+	9.35±1.51	(4.55, 14.16)	8.60±2.26	(1.39, 15.81)	10.56±1.66	(6.31, 14.82)
2		13.73±2.11	(8.30, 19.16)	10.78±1.50	(6.91, 14.63)	-	-
6		13.72±2.31	(7.78, 19.66)	10.79±1.74	(6.33, 15.26)	10.92±1.32	(7.53, 14.31)
10		12.65±2.34	(6.63, 18.66)	10.53±1.47	(6.74, 14.32)	12.43±1.55	(8.44, 16.42)
14		-	-	-	-	11.58±2.34	(5.56, 17.59)
Pre (0)	CD3+CD4+CM+	3.24±0.44	(1.85, 4.64)	3.64±1.01	(0.42, 6.86)	4.21±0.48	(2.96, 5.45)
2		4.70±0.54	(3.31, 6.09)	4.37±0.68	(2.61, 6.13)	-	-
6		5.29±0.75	(3.36, 7.22)	4.35±0.59	(2.83, 5.87)	4.68±0.59	(3.16, 6.20)
10		4.19±0.46	(3.01, 5.37)	4.21±0.65	(2.54, 5.87)	5.87±0.84	(3.70, 8.04)
14		-	-	-	-	4.73±0.39	(3.73, 5.73)
Pre (0)	CD3+CD4+HLADR+	0.90±0.13	(0.32, 1.48)	0.66±0.10	(0.32, 0.99)	0.50±0.10	(0.23, 0.76)
2		1.16±0.15	(0.78, 1.55)	0.98±0.08	(0.76, 1.20)	-	-
6		1.21±0.29	(0.47, 1.95)	0.95±0.13	(0.61, 1.30)	0.93±0.14	(0.56, 1.29)
10		1.22±0.21	(0.69, 1.75)	0.95±0.06	(0.80, 1.11)	1.09±0.14	(0.74, 1.45)
14		-	-	-	-	0.92±0.21	(0.39, 1.45)
Pre (0)	CD3+CD4+Ki67+	1.55±0.49	(-0.02, 3.13)	1.73±0.48	(0.19, 3.26)	2.10±0.47	(0.90, 3.30)
2		1.78±0.45	(0.62, 2.94)	1.44±0.29	(0.69, 2.19)	-	-
6		2.65±0.61	(1.08, 4.21)	1.92±0.24	(1.29, 2.55)	1.65±0.28	(0.93, 2.37)
10		2.05±0.61	(0.47, 3.63)	1.71±0.32	(0.87, 2.54)	2.32±0.42	(1.24, 3.41)
14		-	-	-	-	1.60±0.20	(1.09, 2.11)
Pre (0)	CD3+CD4+Naive+	5.85±1.37	(1.49, 10.20)	4.78±1.46	(0.14, 9.43)	6.08±1.48	(2.28, 9.88)
2		8.58±1.59	(4.49, 12.66)	5.88±0.94	(3.45, 8.29)	-	-
6		7.96±1.89	(3.11, 12.82)	6.08±1.27	(2.81, 9.35)	5.93±1.21	(2.81, 9.05)
10		7.99±1.91	(3.10, 12.89)	5.94±0.92	(3.58, 8.30)	6.16±1.24	(2.96, 9.36)
14		-	-	-	-	6.23±1.86	(1.45, 11.02)
Pre (0)	CD3+CD4+CD25+	0.28±0.06	(0.08, 0.47)	0.40±0.11	(0.03, 0.77)	0.44±0.08	(0.23, 0.65)
2		0.42±0.05	(0.28, 0.55)	0.47±0.10	(0.20, 0.74)	-	-
6		0.61±0.12	(0.31, 0.92)	0.57±0.16	(0.16, 0.99)	0.54±0.07	(0.34, 0.74)
10		0.47±0.10	(0.22, 0.73)	0.53±0.10	(0.27, 0.78)	0.58±0.11	(0.31, 0.85)
14		-	-	-	-	0.47±0.11	(0.18, 0.76)
Pre (0)	CD3+CD4+EM+	0.26±0.08	(0.01, 0.51)	0.15±0.03	(0.06, 0.25)	0.25±0.08	(0.03, 0.47)
2		0.43±0.11	(0.14, 0.73)	0.51±0.10	(0.25, 0.78)	-	-
6		0.44±0.12	(0.12, 0.76)	0.34±0.05	(0.21, 0.47)	0.31±0.10	(0.06, 0.57)
10		0.44±0.09	(0.20, 0.68)	0.36±0.04	(0.26, 0.45)	0.40±0.12	(0.09, 0.71)
14		-	-	-	-	0.62±0.44	(-0.52, 1.77)
Pre (0)	CD3+CD4+CD69+	0.52±0.17	(-0.22, 1.27)	1.89±1.56	(-3.06, 6.85)	1.45±0.96	(-1.02, 3.91)
2		0.59±0.11	(0.30, 0.89)	0.52±0.08	(0.30, 0.76)	-	-
6		0.59±0.15	(0.20, 0.97)	2.80±2.37	(-3.28, 8.88)	0.53±0.10	(0.28, 0.78)
10		0.67±0.16	(0.25, 1.08)	1.60±1.05	(-1.09, 4.30)	0.56±0.09	(0.34, 0.79)
14		-	-	-	-	0.55±0.13	(0.22, 0.88)
Pre (0)	CD3+CD4+CD62L+	7.77±2.06	(-1.10, 16.63)	5.69±1.52	(0.86, 10.54)	7.15±1.31	(3.77, 10.52)
2		9.73±1.80	(5.10, 14.36)	6.77±0.91	(4.42, 9.11)	-	-
6		9.90±1.98	(4.82, 14.98)	7.15±1.54	(3.20, 11.10)	6.84±1.25	(3.63, 10.05)
10		9.53±2.10	(4.12, 14.94)	7.48±1.05	(4.78, 10.17)	7.98±1.21	(4.88, 11.08)
14		-	-	-	-	6.68±1.32	(3.30, 10.07)
Pre (0)	CD3+CD4+CD38+	0.90±0.32	(-0.49, 2.30)	0.58±0.24	(-0.17, 1.34)	0.97±0.20	(0.46, 1.48)
2		1.13±0.19	(0.65, 1.62)	0.97±0.08	(0.75, 1.18)	-	-
6		1.27±0.27	(0.58, 1.95)	0.89±0.15	(0.49, 1.29)	0.84±0.14	(0.47, 1.20)
10		1.09±0.17	(0.65, 1.52)	0.92±0.03	(0.85, 0.99)	1.23±0.19	(0.73, 1.73)
14		-	-	-	-	0.71±0.11	(0.43, 0.99)

*Absolute values x 10^2/µl of blood

Supplemental Table S3: Mean ± Standard Error (SE) and Confidence Interval (CI) of different subsets of CD8+ T cells at different housing groups*

Time points In Weeks	Cell Subsets	GH-SH		GH-PH		GH-PH-SH	
		**Mean ± SE**	**Cl (95%)**	**Mean ± SE**	**Cl (95%)**	**Mean ± SE**	**Cl (95%)**
Pre (0)	CD3+CD8+	6.14±1.05	(2.79, 9.48)	3.88±1.03	(0.59, 7.16)	6.05±0.79	(4.02, 8.09)
2		10.45±1.70	(6.08, 14.82)	8.55±1.59	(4.47, 12.63)	-	-
6		11.09±2.39	(4.94, 17.25)	7.58±1.69	(3.24, 11.93)	7.42±1.08	(4.65, 10.18)
10		9.14±1.88	(4.31, 13.96)	6.53±1.03	(3.89, 9.18)	10.73±1.68	(6.41, 15.05)
14		-	-	-	-	8.60±2.66	(1.77, 15.43)
Pre (0)	CD3+CD8+CM+	1.20±0.16	(0.70, 1.70)	1.02±0.17	(0.47, 1.57)	1.79±0.43	(0.68, 2.90)
2		2.45±0.40	(1.42, 3.48)	2.01±0.29	(1.25, 2.77)	-	-
6		2.79±0.70	(0.98, 4.59)	2.32±0.57	(0.84, 3.79)	2.02±0.36	(1.08, 2.95)
10		2.05±0.33	(1.19, 2.90)	1.68±0.28	(0.96, 2.39)	3.02±0.55	(1.62, 4.42)
14		-	-	-	-	1.91±0.31	(1.12, 2.71)
Pre (0)	CD3+CD8+HLADR+	1.12±0.28	(-0.09, 2.33)	1.12±0.10	(0.81, 1.43)	1.17±0.64	(-0.47, 2.82)
2		1.55±0.32	(0.72, 2.38)	2.43±0.53	(1.07, 3.78)	-	-
6		1.79±0.52	(0.46, 3.12)	2.42±0.78	(0.40, 4.43)	0.72±0.09	(0.50, 0.96)
10		1.03±0.16	(0.60, 1.45)	1.67±0.21	(1.14, 2.21)	1.46±0.27	(0.76, 2.17)
14		-	-	-	-	0.84±0.30	(0.06, 1.62)
Pre (0)	CD3+CD8+Ki67+	1.22±0.30	(0.25, 2.19)	0.93±0.24	(0.17, 1.68)	1.14±0.19	(0.66, 1.62)
2		1.77±0.54	(0.38, 3.15)	1.40±0.41	(0.35, 2.46)	-	-
6		3.66±1.01	(1.07, 6.26)	2.71±0.93	(0.31, 5.11)	1.43±0.40	(0.41, 2.46)
10		2.11±0.49	(0.83, 3.38)	1.21±0.45	(0.45, 1.97)	3.29±0.60	(1.73, 4.84)
14		-	-	-	-	1.32±0.23	(0.73, 1.91)
Pre (0)	CD3+CD8+Naive+	2.56±0.72	(0.27, 4.85)	1.30±0.32	(0.27, 2.32)	2.39±0.71	(0.56, 4.21)
2		4.13±0.87	(1.89, 6.36)	2.11±0.23	(1.50, 2.71)	-	-
6		3.59±1.16	(0.61, 6.58)	1.88±0.30	(1.09, 2.67)	2.91±0.77	(0.94, 4.89)
10		3.34±0.94	(0.92, 5.76)	1.87±0.36	(0.95, 2.80)	3.32±1.01	(0.72, 5.93)
14		-	-	-	-	3.07±1.21	(-0.04, 6.19)
Pre (0)	CD3+CD8+CD25+	0.31±0.05	(0.14, 0.49)	0.35±0.05	(0.14, 0.48)	0.39±0.05	(0.26, 0.52)
2		0.51±0.08	(0.31, 0.72)	0.42±0.08	(0.31, 0.72)	-	-
6		0.52±0.09	(0.30, 0.74)	0.39±0.09	(0.29, 0.74)	0.38±0.03	(0.30, 0.47)
10		0.47±0.09	(0.24, 0.70)	0.35±0.09	(0.23, 0.70)	0.64±0.13	(0.32, 0.96)
14		-	-	-	-	0.39±0.09	(0.14, 0.64)
Pre (0)	CD3+CD8+EM+	2.38±0.68	(0.23, 4.54)	1.55±0.57	(-0.28, 3.37)	1.84±0.31	(1.05, 2.63)
2		3.87±0.78	(1.87, 5.88)	4.41±1.22	(1.27, 7.56)	-	-
6		4.50±1.05	(1.81, 7.19)	3.37±1.05	(0.66, 6.08)	2.49±0.30	(1.73, 3.25)
10		3.74±0.78	(1.74, 5.75)	2.98±0.68	(1.22, 4.74)	4.37±0.78	(2.37, 6.36)
14		-	-	-	-	3.60±1.41	(-0.02, 7.23)
Pre (0)	CD3+CD8+CD69+	1.73±0.15	(1.07, 2.39)	2.15±0.68	(-0.02, 4.32)	0.82±0.11	(0.54, 1.10)
2		2.43±0.49	(1.17, 3.68)	4.50±1.43	(0.83, 8.17)	-	-
6		1.91±0.51	(0.60, 3.21)	3.07±0.78	(1.07, 5.08)	1.17±0.18	(0.72, 1.62)
10		1.75±0.49	(0.49, 3.00)	2.85±0.61	(1.28, 4.41)	1.93±0.37	(0.97, 2.88)
14		-	-	-	-	1.65±0.69	(-0.12, 3.42)
Pre (0)	CD3+CD8+CD62L+	3.72±0.99	(-0.58, 8.02)	4.55±1.06	(1.19, 7.91)	3.19±0.75	(1.27, 5.11)
2		5.65±1.12	(2.76, 8.54)	7.03±0.95	(4.57, 9.49)	-	-
6		5.91±1.48	(2.11, 9.71)	6.86±0.92	(4.49, 9.22)	3.85±0.75	(1.91, 5.79)
10		4.92±1.19	(1.86, 7.98)	7.34±1.14	(4.42, 10.27)	5.12±1.12	(2.24, 8.00)
14		-	-	-	-	3.70±0.91	(1.36, 6.04)
Pre (0)	CD3+CD8+CD38+	0.71±0.08	(0.32, 1.09)	0.99±0.32	(-0.02, 2.02)	0.56±0.10	(0.31, 0.81)
2		1.50±0.28	(0.76, 2.23)	2.06±0.40	(1.02, 3.10)	-	-
6		1.12±0.27	(0.42, 1.81)	1.87±0.53	(0.52, 3.22)	0.66±0.12	(0.37, 0.96)
10		0.79±0.18	(0.32, 1.27)	1.70±0.29	(0.95, 2.45)	1.13±0.23	(0.53, 1.73)
14		-	-	-	-	0.78±0.29	(0.04, 1.53)

* Absolute values x 10^2/µl of blood

Supplemental Table S4: Mean ± Standard Error (SE) and Confidence Interval (CI) of different subsets of CD20+ B cells at different housing groups*

Time points In Weeks	Cell Subsets	GH-SH		GH-PH		GH-PH-SH	
		**Mean ± SE**	**Cl (95%)**	**Mean ± SE**	**Cl (95%)**	**Mean ± SE**	**Cl (95%)**
Pre (0)	CD20+	19.36±5.28	(2.56, 36.16)	14.54±3.43	(3.62, 25.46)	13.43±2.16	(7.88, 18.99)
2		21.72±4.44	(10.30, 33.14)	18.62±4.87	(6.11, 31.13)	-	-
6		20.48±3.68	(11.00, 29.95)	16.52±3.83	(6.11, 31.13)	13.75±1.61	(9.61, 17.90)
10		21.08±2.79	(13.89, 28.27)	17.17±4.83	(6.67, 26.37)	15.28±2.12	(9.83, 20.73)
14		-	-	-	(4.76, 29.58)	14.69±2.74	(7.64, 21.74)
Pre (0)	CD20+CD25+	0.90±0.17	(0.36, 1.44)	0.62±0.17	(0.07, 1.18)	0.56±0.09	(0.32, 0.81)
2		1.41±0.40	(0.38, 2.43)	0.97±0.23	(0.37, 1.56)		
6		1.32±0.29	(0.58, 2.06)	1.16±0.27	(0.47, 1.85)	0.89±0.14	(0.53, 1.25)
10		1.11±0.12	(0.79, 1.43)	1.10±0.31	(0.31, 1.89)	1.01±0.19	(0.51, 1.51)
14		-	-	-	-	0.86±0.20	(0.33, 1.38)
Pre (0)	CD20+CD38+	1.18±0.39	(-0.48, 2.84)	0.77±0.20	(0.13, 1.41)	0.79±0.15	(0.40, 1.18)
2		2.33±0.55	(0.92, 3.75)	1.03±0.23	(0.42, 1.63)	-	-
6		2.04±0.31	(1.24, 2.85)	1.25±0.26	(0.58, 1.92)	0.85±0.19	(0.35, 1.34)
10		2.43±0.61	(0.86, 3.99)	1.32±0.27	(0.63, 2.00)	1.10±0.27	(0.42, 1.78)
14		-	-	-	-	0.99±0.21	(0.45, 1.53)
Pre (0)	CD20+CD69+	1.57±0.29	(0.34, 2.80)	1.07±0.17	(0.53, 1.60)	0.93±0.18	(0.46, 1.39)
2		1.85±0.44	(0.71, 2.98)	1.49±0.34	(0.62, 2.36)	-	-
6		1.78±0.28	(1.06, 2.50)	1.45±0.26	(0.78, 2.12)	1.20±0.22	(0.64, 1.76)
10		1.74±0.24	(1.13, 2.36)	1.49±0.36	(0.55, 2.42)	1.30±0.22	(0.74, 1.86)
14		-	-	-	-	1.26±0.22	(0.70, 1.81)
Pre (0)	CD20+CD62L+	3.87±0.55	(1.49, 6.25)	2.61±0.95	(-0.41, 5.63)	1.70±0.49	(0.44, 2.96)
2		6.44±1.52	(2.54, 10.34)	4.73±1.35	(1.26, 8.20)		
6		5.47±1.48	(1.65, 9.29)	3.28±0.98	(0.77, 5.79)	2.68±0.43	(1.58, 3.79)
10		5.46±0.98	(2.94, 7.93)	3.61±0.92	(1.25, 5.97)	3.04±0.62	(1.44, 4.64)
14		-	-	-	-	2.92±0.34	(2.03, 3.81)
Pre (0)	CD20+Ki67+	3.18±1.41	(-1.31, 7.68)	2.74±1.09	(-0.73, 6.22)	1.53±0.23	(0.94, 2.11)
2		2.89±0.80	(0.82, 4.96)	2.57±0.79	(0.51, 4.62)	-	-
6		4.65±0.41	(3.59, 5.71)	3.84±0.67	(2.12, 5.56)	2.10±0.20	(1.59, 2.62)
10		3.14±0.66	(1.44, 4.84)	2.31±0.75	(0.38, 4.24)	3.40±0.55	(1.99, 4.81)
14		-	-	-	-	1.98±0.18	(1.53, 2.44)

* Absolute values x 10^2/µl of blood
